# IgG‐Bridging–Seeded Synergistic Aggregation of SARS‐CoV‐2 Spikes Underlies Potent Neutralization by a Low‐Affinity Antibody

**DOI:** 10.1002/advs.202517192

**Published:** 2025-12-07

**Authors:** Niannian Lv, Peng Chen, Xiaobin Dai, Hu Xu, Ziheng Li, Zelin Shan, Jinqian Li, Fenglin Guo, Yuanfang Chen, Jiayi Li, Yiqian Huang, Guizhi Dong, Yifan Jiang, Liang Chen, Xuanyu Nan, Hanjun Zhao, Kang Zhang, Shilong Fan, Yuanchen Dong, Dongsheng Liu, Xinquan Wang, Deli Huang, Xiaojing Pan, Chunying Chen, Zhihua Liu, Li‐Tang Yan, Qi Zhang, Linqi Zhang, Yuliang Zhao, Yuhe Renee Yang

**Affiliations:** ^1^ National Cancer Center/National Clinical Research Center for Cancer/Cancer Hospital Chinese Academy of Medical Sciences and Peking Union Medical College Beijing 100021 China; ^2^ Laboratory of Nanosystem and Hierarchical Fabrication National Center for Nanoscience and Technology Beijing 100190 China; ^3^ Research Unit of Nanoscience and Technology Chinese Academy of Medical Sciences Beijing 100730 China; ^4^ Comprehensive AIDS Research Center Pandemic Research Alliance Unit Center for Infection Biology School of Basic Medical Sciences Tsinghua University Beijing 100084 China; ^5^ State Key Laboratory of Chemical Engineering and Low‐carbon Technology Department of Chemical Engineering and Low‐carbon Technology Tsinghua University Beijing 100084 China; ^6^ CAS Key Laboratory for Biological Effects of Nanomaterials and Nanosafety National Center for Nanoscience and Technology Chinese Academy of Sciences Beijing 100190 China; ^7^ College of Ecology Lanzhou University Lanzhou 730000 China; ^8^ University of Chinese Academy of Sciences Beijing 100049 China; ^9^ The Salk Institute of Biological Sciences La Jolla CA 92037 USA; ^10^ The Ministry of Education Key Laboratory of Protein Science Beijing Frontier Research Center for Biological Structure School of Life Sciences Tsinghua University Beijing 100084 China; ^11^ CAS Key Laboratory of Colloid Interface and Chemical Thermodynamics Institute of Chemistry Chinese Academy of Sciences Beijing 100190 China; ^12^ MOE Engineering Research Center of Advanced Rare Earth Materials Department of Chemistry Tsinghua University Beijing 100084 China; ^13^ Shanghai Institute of Infectious Disease and Biosecurity Shanghai Medical College Fudan University Shanghai 200032 China; ^14^ Centre for Virology Vaccinology and Therapeutics Department of Microbiology Li Ka Shing Faculty of Medicine The University of Hong Kong Hong Kong SAR 999077 China; ^15^ The technology center for protein science Tsinghua University Beijing 100084 China; ^16^ Department of Applied Biology and Chemical Technology The Hong Kong Polytechnic University Hong Hum Kowloon 999077 Hong Kong; ^17^ PolyU Shenzhen Research Institute Shenzhen 518057 China; ^18^ Zhejiang Provincial Key Laboratory for Cancer Molecular Cell Biology Life Sciences Institute Zhejiang University Hangzhou 310058 China; ^19^ Institute of Bio‐Architecture and Bio‐Interactions Shenzhen Medical Academy of Research and Translation Shenzhen Guangdong 518107 China; ^20^ CAS Key Laboratory for Biomedical Effects of Nanomaterials and Nanosafety and CAS Center for Excellence in Nanoscience Beijing 100190 China; ^21^ Department of Gastrointestinal Oncology Key Laboratory of Carcinogenesis and Translational Research (Ministry of Education) Beijing 100142 China; ^22^ Institute of Biopharmaceutical and Health Engineering Tsinghua Shenzhen International Graduate School Tsinghua University Shenzhen 518055 China; ^23^ Institute of Biomedical Health Technology and Engineering Shenzhen Bay Laboratory Shenzhen 518132 China

**Keywords:** full‐length IgG, low‐affinity, potent neutralization, multivalent aggregation, virion‐like nanoparticles

## Abstract

Mechanistic studies of viral neutralization typically prioritize high‐affinity antibodies, relegating low‐affinity binders to the sidelines. P5‑1C8, a Class 1 SARS‐CoV‐2 antibody that exemplifies this underexplored “low‑affinity yet high‑potency” phenotype is reported, retaining strong neutralization of Omicron JN.1 despite markedly weakened trimer binding (K_D_ = 225 nM; IC_50_ = 0.06 nM). Structural and biophysical analyses reveal that P5‐1C8 engages WT and BA.1 spikes through canonical intra‐spike bivalency, but with JN.1 it induces aggregation. Using virion‐like nanoparticles displaying multiple spikes, it is shown that IgG remains bound with no detectable dissociation and triggers pronounced aggregation. Coarse‐grained molecular dynamics delineate the stepwise pathway in which weak IgG‐spike contacts seed aggregation via transient inter‐spike bridging. Together, these findings establish the first mechanistic framework demonstrating how weak‐binding antibodies can nonetheless achieve potent neutralization through higher‐order aggregation, thereby expanding the conceptual landscape of antibody function and opening new directions for antibody evaluation and design.

## Graphical abstract

Low‐affinity antibodies are frequently disregarded in discovery pipelines. This work reports P5‐1C8, a Class 1 SARS‐CoV‐2 antibody with weak trimer binding (K_D_‐to‐IC_50_ > 3700‐fold) yet potent neutralization of Omicron JN.1. Structural, biophysical, functional, and coarse‐grained simulations collectively demonstrate that transient inter‐spike IgG bridging seeds higher‐order aggregation, which in turn drives neutralization and provides a mechanistic framework.

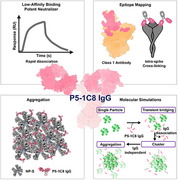

## 1 Introduction

Neutralizing antibodies are central to humoral immunity against viral infections.^[^ ^1^ ^]^ They employ diverse mechanisms, ranging from blocking viral attachment by competing with receptor binding,^[^ ^2^ ^]^ inducing spike conformational changes,^[^ ^3^ ^]^ or promoting virion aggregation;^[^ ^4^, ^5^ ^]^ to interfering with membrane fusion,^[^ ^6^ ^]^ endosomal cleavage, or receptor engagement after attachment;^[^ ^7^, ^8^ ^]^ to restricting viral spread by suppressing progeny release^[^ ^9^ ^]^ or preventing direct cell‐to‐cell transmission.^[^ ^10^, ^11^ ^]^ Despite their mechanistic diversity, all these processes converge on a common initiating event—binding. In the case of severe acute respiratory syndrome‐coronavirus‐2 (SARS‐CoV‐2), numerous studies have focused on antibodies with high‐affinity and potent neutralization,^[^ ^12^, ^13^, ^14^, ^15^ ^]^ with general expectation that binding affinity—or more accurately, avidity—correlates with neutralization.^[^ ^16^, ^17^ ^]^ Using a combined dataset of equilibrium dissociation constants (K_D_) and neutralization potencies (IC_50_) for coronavirus‐related antibodies, compiled from the Ab‐CoV database (reported up to 2022; https://web.iitm.ac.in/bioinfo2/ab‐cov/covabseq/),^[^ ^18^ ^]^ and supplemented with additional data published after 2022, we noted that affinity and avidity measurements are almost exclusively derived from binding to monomeric receptor‐binding domains (RBDs), rather than to trimeric spike proteins. While this approach enables the distinction between Fab‐RBD and IgG‐RBD interactions, which often reveal affinity or avidity differences ranging from several‐fold to several hundred‐fold. It overlooks key structural features of the native spike, such as dynamic “up” and “down” RBD conformations,^[^ ^19^, ^20^, ^21^ ^]^ as well as critical factors of bivalent IgG binding, including Fab approach angles,^[^ ^22^ ^]^ post‐binding stoichiometry,^[^ ^23^, ^24^, ^25^ ^]^ and multivalent binding modes.^[^ ^14^, ^22^, ^26^ ^]^ In contrast to the vast majority of studies that rely on monomeric domains, only a handful of reports to date have examined the binding of Fabs and full‐length IgGs to the intact spike trimer.^[^ ^22^, ^27^, ^28^, ^29^ ^]^ From these limited trimer‐based studies, a consistent observation has emerged: high‐affinity Fabs (sub‐nanomolar) confer potent neutralization in both Fab and IgG formats, whereas moderate‐affinity Fabs (tens of nanomolar) lose neutralizing activity in Fab fragment form but retain potency as full‐length IgGs due to bivalent engagement.^[^ ^22^ ^]^ Rare exceptions, such as COVA1‐03^[^ ^13^ ^]^ and COVA1‐25 ^[^ ^13^ ^]^ display undetectable apparent avidity yet maintain strong neutralizing activity. These antibodies have received little mechanistic investigation, likely due to their rarity, the prevailing emphasis on high‐affinity candidates in antibody discovery pipelines,^[^ ^12^, ^13^, ^14^, ^15^ ^]^ and a methodological disconnect where binding is typically measured using monomeric RBDs or Fab fragments, whereas neutralization is measured using full‐length IgGs against pseudovirus or live virus.^[^ ^30^ ^]^

To elucidate the mechanism of antibody‐mediated neutralization, structural biology is widely employed to map epitopes, define binding interactions, and reveal the modes of neutralization.^[^ ^31^ ^]^ Most studies to date have focused on Fab‐RBD complexes using X‐ray crystallography^[^ ^25^, ^32^, ^33^, ^34^, ^35^, ^36^, ^37^, ^38^ ^]^ or Fab‐spike complexes using cryo‐electron microscopy (cryo‑EM),^[^ ^39^, ^40^, ^41^, ^42^, ^43^, ^44^, ^45^, ^46^, ^47^, ^48^, ^49^, ^50^ ^]^ approaches that provide high‐resolution insight into epitope targeting but fail to capture avidity effects and Fc‐mediated contributions.^[^ ^51^ ^]^ In contrast, analyses of full‐length W328‐6H2 IgG have revealed distinct, variant‐specific higher‐order architectures that directly affect its neutralizing capability.^[^ ^27^ ^]^ Such higher‐order IgG‐spike assemblies can reveal previously inaccessible dimensions of antibody function that are not captured by conventional structural approaches. For instance, while antibody P17 was previously known to neutralize SARS‐CoV‐2 by blocking ACE2 binding and inhibiting membrane fusion,^[^ ^52^ ^]^ and S309 was recognized for targeting the conserved N343 glycan and exhibiting enhanced Fc‐dependent effector functions.^[^ ^28^ ^]^ However, visualization of full‐length IgG interacting with native spike trimers on the viral surface has uncovered previously unrecognized mechanisms. For P17, cryo‐electron tomography revealed that bivalent IgG assembles spike trimers into linear multimers, likely generating mechanical tension that induces S1 shedding—an effect not apparent from Fab‐based or isolated trimer studies.^[^ ^53^ ^]^ Similarly, for S309, higher‐order pentameric and hexameric lattice structures were observed,^[^ ^53^ ^]^ providing a structural basis for enhanced complement activation,^[^ ^54^ ^]^ antibody‐dependent cell cytotoxicity (ADCC), antibody‐dependent cellular phagocytosis (ADCP), and FcγRIIa signaling.^[^ ^28^, ^53^ ^]^ Together, these findings demonstrate that Fab‐based structures alone cannot capture the full scope of antibody function. A comprehensive understanding of neutralization mechanisms requires multiscale structural analyses—from Fab and full‐length IgG, and from RBD to spike trimer to virion—combined with diverse biophysical and functional assays.

In this work, we describe P5‐1C8, a broadly neutralizing antibody isolated from a convalescent individual infected with the ancestral SARS‐CoV‐2 strain. Despite exhibiting markedly reduced binding avidity to the Omicron subvariant JN.1 (K_D_ = 225 nM), P5‐1C8 retains potent neutralization activity (IC_50_ = 0.06 nM), resulting in a >3,700‐fold K_D_‐to‐IC_50_ disparity. X‑ray crystallography mapped its epitope to the receptor‑binding motif (RBM), confirming its Class 1 category, which almost invariably employs the canonical intra‐spike bivalent binding mode.^[^ ^22^ ^]^ Negative‑stain and single‐particle cryo‐EM revealed that P5‐1C8 adopts the standard intra‐spike bivalent geometry on both wild‐type (WT) and Omicron BA.1 spikes, in line with prior structures.^[^ ^22^ ^]^ In contrast, no such binding mode was detected with JN.1; instead, P5‐1C8 promoted extensive aggregation. This behavior was corroborated using virion‐like nanoparticles displaying multiple spikes, where multivalent presentation abolished measurable IgG dissociation and drove large‐scale aggregation. Coarse‐grained molecular dynamics further delineated the process: on WT nanoparticles, P5‐1C8 engaged in high‐avidity intra‐ and inter‐spike binding mode, whereas weaker binding to JN.1 spikes seeded transient assemblies, which initiate the stable aggregation. Together, these results reveal that low‐affinity antibodies—even those ineffective in monovalent assays—can exert potent neutralization by a previously underappreciated mechanism of higher‐order assembly, and expand the conceptual framework of antibody function and underscore the need to systematically include such antibodies in mechanistic studies, therapeutic discovery, and vaccine evaluation.

## 2 Results

### 2.1 Neutralization Potency, Binding Affinity, and Epitope Mapping of Antibody P5‐1C8

**Figure 1:**
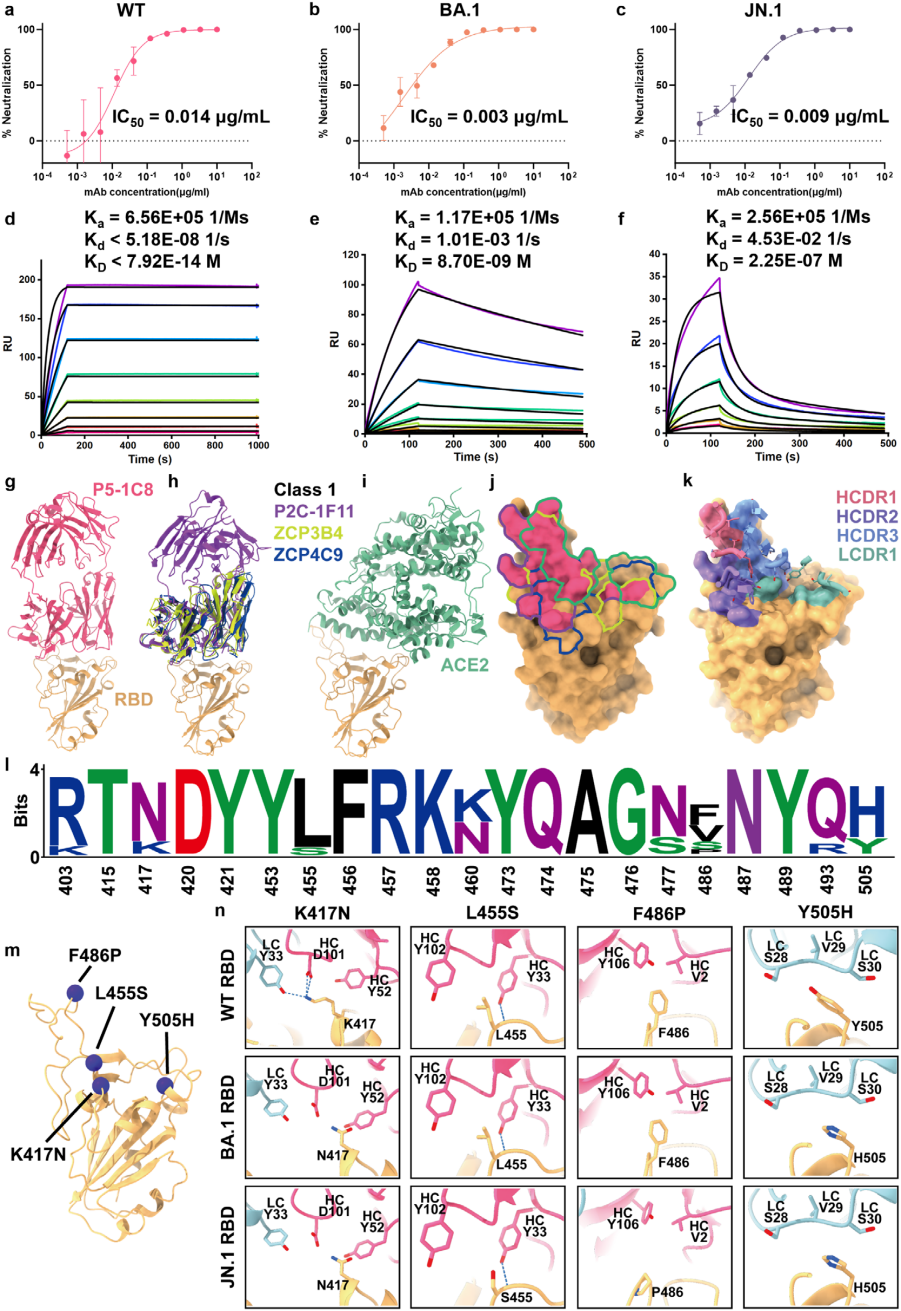
Neutralization potency, binding affinity, and epitope mapping of antibody P5‐1C8. a‐c) Neutralization c0075rves of P5‐1C8 IgG against SARS‐CoV‐2 WT (a), BA.1 (b), and JN.1 (c) variants. Data were obtained from two independent experiments, each performed with two technical replicates. d–f) Binding kinetics between P5‐1C8 IgG and the spike trimer of SARS‐CoV‐2 WT (d), BA.1 (e), and JN.1 (f), measured by surface plasmon resonance (SPR). Spike trimers were immobilized on a nitrilotriacetic acid (NTA) sensor chip, and serial dilutions of P5‐1C8 IgG were flowed through the system. Colored lines represent experimentally measured sensorgrams. Black lines show the best‐fit curves based on experimental data. The calculated association rate (K_a_), dissociation rate (K_d_), and equilibrium dissociation constant (K_D_) for each antibody‐spike pair are indicated. The dissociation rate constant of P5‐1C8 IgG for WT spike should be interpreted with caution, as it is near the detection limit of the instrument. All results were confirmed in three independent experiments. g) Overall structure of the complex between SARS‐CoV‐2 WT RBD (soft orange) and P5‐1C8 Fab (pink). h) Superimposition of three Fab‐RBD complexes: P2C‐1F11 (dark violet), ZCP3B4 (moderate yellow), and ZCP4C9 (blue). i) Crystal structure of WT RBD (soft orange) in complex with ACE2 (green), panels g‐i show the spatial relationships of all four Fabs relative to the ACE2 binding site. j) Binding footprints of the four Fabs and ACE2 on the SARS‐CoV‐2 RBD. Pink, dark violet, moderate yellow, blue, and green denote the footprints of P5‐1C8 Fab, P2C‐1F11 Fab, ZCP3B4 Fab, ZCP4C9 Fab, and ACE2, respectively. k) Footprint of RBD‐contacting residues on antibody CDR loops, with key amino acids in HCDR1‐3 and LCDR1. CDR positions are annotated according to IMGT numbering. l) Sequence conservation analysis on residues bound by P5‐1C8. These logo plots show the conservation of P5‐1C8 epitopes from SARS‐CoV‐2 WT, Beta, Delta, and Omicron (BA.1, BA.2.75, BA.5, BF.7, BQ.1, BQ.1.1, XBB, XBB.1, and JN.1) variants. m) Structure of the SARS‐CoV‐2 WT RBD, with key P5‐1C8 Fab‐binding residues highlighted as violet spheres. n) The interactions between P5‐1C8 Fab and non‐conserved residues in the RBD are affected by strain‐specific mutations during viral evolution. Shown are the structural comparisons of P5‐1C8 Fab interactions with WT RBD (upper panel) and Omicron BA.1 RBD (middle panel), and JN.1 RBD (lower panel) at positions K417N, L455S, F486P, and Y505H. Contacting residues are depicted as sticks, and hydrogen bonds or salt bridges are indicated by dashed lines.

As detailed in our previously published work,^[^ ^32^ ^]^ we isolated a broadly neutralizing antibody, P5‐1C8, from a convalescent individual (P5) following ancestral SARS‐CoV‐2 infection. P5‐1C8 demonstrated exceptional neutralizing potency against wild‐type (WT) SARS‐CoV‐2 virus (IC_50_ = 0.014 µg/mL), and maintained strong neutralizing activity across a panel of major SARS‐CoV‐2 variants, including Beta, Delta, BA.1, BA.2.75, BA.5, BF.7, BQ.1, BQ.1.1, XBB, XBB.1, and JN.1, with IC_50_ values ranging from 0.003 to 0.042 µg/mL (**Figure** **1**a‐c; Figure S1, Supporting Information). To investigate the mechanistic basis underlying the broad and potent neutralizing activity of P5‐1C8, we first characterized the binding kinetics of full‐length P5‐1C8 IgG using surface plasmon resonance (SPR), with His‐tagged trimeric spike proteins immobilized on nitrilotriacetic acid (NTA) sensor chips. P5‐1C8 displayed extremely high avidity for WT spike trimer (K_D_ < 1 pM, Figure 1d), and retained strong binding for BA.1 spike trimer (K_D_ ≈ 8.7 nM, Figure 1e). Remarkably, however, its binding to JN.1 spike trimer was substantially reduced, with a K_D_ of approximately 225 nM—nearly three orders of magnitude weaker than BA.1 and over five orders weaker than WT (Figure 1f). While the association rate constant (K_a_) remained comparable across WT, BA.1, and JN.1 spikes, the dissociation rate constant (K_d_) for JN.1 was elevated by approximately six orders of magnitude compared to WT spike. Despite this markedly reduced binding avidity, P5‐1C8 retained potent neutralization against JN.1, with an IC_50_ of 0.009 µg/mL (Figure 1c). The striking disconnect between binding avidity and neutralization potency—a ≈3750‐fold divergence between K_D_ and IC_50_—represents a profound decoupling of antigen recognition from antiviral efficacy. This observation stands in sharp contrast to prevailing models of antibody‐mediated neutralization, in which affinity or avidity is generally predictive of functional potency (Table S1, Supporting Information).

Given the striking disconnect between binding avidity and neutralization potency, we next sought to define the structural basis of P5‐1C8 recognition through high‐resolution epitope mapping. To this end, we determined the crystal structure of the P5‐1C8 Fab in complex with the wild‐type SARS‐CoV‐2 RBD at 2.39‐Å resolution (Figure 1g; Figure S2, Supporting Information). Structural alignment with three previously reported Fab‐RBD complexes revealed that P5‐1C8 shares a nearly identical binding pose with P2C‐1F11,^[^ ^35^ ^]^ ZCP3B4,^[^ ^42^ ^]^ and ZCP4C9,^[^ ^42^ ^]^ targeting the top face of RBD, mimicking the binding mode of ACE2 (Figure 1h,i).^[^ ^35^ ^]^ All four antibodies adopt a similar footprint as well as orientation characteristic of class 1 RBD‐targeting antibodies (Figure 1j; Figure S3, Supporting Information). The P5‐1C8 paratope comprises 21 residues, with 17 contributed by the heavy chain (6 from HCDR1, 5 from HCDR2, 5 from HCDR3) and 4 from the light chain (all from LCDR1), burying a total surface area of ≈1003.7 Å^2^ (Figure 1k). The interface includes 15 hydrogen bonds and two salt bridges (Table S4, Supporting Information), and exhibits substantial epitope overlap with P2C‐1F11 (21 shared residues), ZCP3B4 (18), ZCP4C9 (19), and ACE2 (10) (Figure S3, Supporting Information), confirming that P5‐1C8 targets a conserved class 1 epitope. To assess conservation, we performed multiple sequence alignments across variants of concern (VOCs; Figure S4, Supporting Information), revealing that 8 of the 21 contact residues are mutated in at least one variant (Figure 1l). Structural analysis of the P5‐1C8‐RBD interface in WT, BA.1, and JN.1 showed that four substitutions (R403K, N460K, S477N, Q493R) caused minimal disruption (Figure S5, Supporting Information), whereas K417N, L455S, F486P, and Y505H significantly altered key interactions (Figure 1m,n). For example, K417N abolishes cation‐π and salt‐bridge interactions;^[^ ^55^ ^]^ L455S increases flexibility and weakens hydrophobic contacts;^[^ ^56^ ^]^ F486P disrupts π‐π stacking and destabilizes local structure;^[^ ^57^, ^58^ ^]^ and Y505H impairs both hydrophobic and aromatic interactions.^[^ ^59^ ^]^ These changes collectively contribute to the observed stepwise reduction in binding avidity from WT to BA.1 to JN.1, as reflected in SPR measurements. Although structural and kinetic analyses align in defining the epitope and affinity/avidity landscape, they cannot account for the potent neutralizing activity observed against JN.1, suggesting that P5‐1C8 may engage in an alternative or auxiliary neutralization mechanism beyond conventional receptor blockade.

### 2.2 Multivalent Binding of Full‐Length P5‐1C8 IgG to WT, BA.1, and JN.1 Spike Trimers

**Figure 2:**
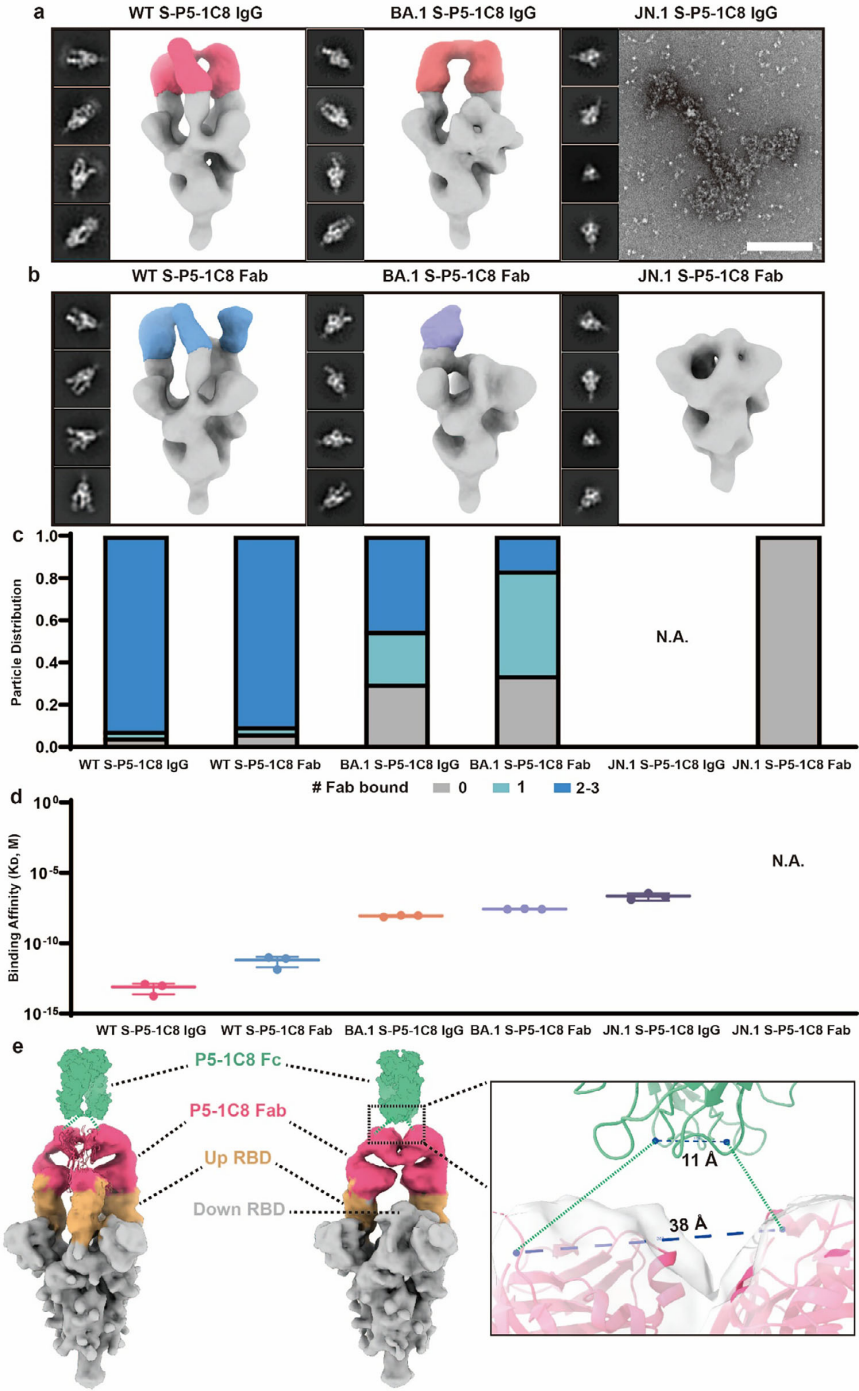
Multivalent binding analysis of antibody P5‐1C8 to WT, BA1, and JN1 spike trimers. a) Representative 2D class averages and side views of composite 3D reconstructions from ns‐EM of P5‐1C8 IgG in complex with spike proteins from SARS‐CoV‐2 WT (pink), BA.1(red) and JN.1, at a 3:1 molar ratio (IgG/Spike). In the JN1 S‐P5‐1C8 IgG complex, particles do not form well‐defined complexes but instead aggregate upon binding. Scale bar: 200 nm. b) Representative 2D class averages and side views of composite 3D reconstructions from ns‐EM of P5‐1C8 Fab in complex with spike proteins from SARS‐CoV‐2 WT (blue), BA.1(purple) and JN.1, at a 3:1 molar ratio (Fab/Protomer). No binding is observed in the JN.1 S‐P5‐1C8 Fab complex. c) Semi‐quantitative epitope occupancy analysis derived from ns‐EM shown in (a) and (b), showing the proportion of spike trimers bound by 0, 1, 2–3 Fabs (gray, light cyan, and blue, respectively). Data correspond to the S‐P5‐1C8 IgG (a) and S‐P5‐1C8 Fab (b) complexes for SARS‐CoV‐2 WT, BA.1, and JN.1, as indicated for each participant on the x‐axis. d) Binding affinity values obtained from SPR assays of P5‐1C8 IgG and Fab against SARS‐CoV‐2 WT, BA.1, and JN.1 spikes. The binding of P5‐1C8 Fab to JN.1 spike was not detectable. N.A., not available. All experiments were performed independently in triplicate. e) Cryo‐EM map of the SARS‐CoV‐2 WT spike in complex with P5‐1C8 IgG. The reconstruction reveals two binding stoichiometries: a symmetric complex (1.5 IgG bound) and an asymmetric complex (one IgG bound). Up RBDs, down RBD, and P5‐1C8 Fabs are shown in yellow, grey, and pink, respectively. The Fc region of P5‐1C8, shown in green, was modeled using AlphaFold 3 predictions based on the P5‐1C8 Fc sequence. The absence of Fc density in the WT S‐P5‐1C8 IgG reconstruction is likely due to intrinsic hinge‐mediated flexibility of the IgG molecule.^[^ ^29^ ^]^ The zoomed‐in view on the right highlights the structural details within the black dashed box. The two CH1 C‐termini of the Fabs are separated by 38 Å, whereas the N‐termini of the Fc heavy chains are spaced 11 Å apart.

To dissect how P5‐1C8 engages SARS‐CoV‐2 spike trimers from the structural perspective, we employed negative‐stain electron microscopy (ns‐EM) to characterize the global structure of P5‐1C8 both in full‐length IgG and Fab fragments in complex with WT, BA.1, and JN.1 spikes (**Figure** **2**a,b; Figure S6, Supporting Information). We grouped the resulting 2D averages based on the number of Fab molecules bound per spike trimer and quantified particle distributions within each class to estimate binding occupancy (Figure 2c), which was subsequently correlated with SPR‐derived binding affinities (Figure 2d; Figure S7, Supporting Information). Upon binding to WT spike, both full‐length IgG and Fab forms of P5‐1C8 exhibited high occupancy, engaging up to three RBDs per trimer (Figure 2a,b). Over 95% of IgG‐spike particles showed 2–3 bound Fabs in 2D class averages, with full occupancy confirmed by 3D reconstruction; Fab displayed >93% occupancy with 3 Fabs per trimer (Figure 2a–c). On average, IgG bound 1.5 molecules per trimer, while Fab saturated all three RBDs. Both forms exhibited picomolar‐range KD values (Figure 2d) and potent neutralization, with IC_50_ values of 0.014 µg/mL for IgG (Figure 1a) and 0.018 µg/mL for Fab (Figure S7a, Supporting Information), consistent with previously reported high‐affinity class 1 antibodies.^[^ ^22^ ^]^

In contrast to WT, P5‐1C8 exhibited reduced binding to the BA.1 spike. Ns‐EM analysis showed a decrease in IgG occupancy to ≈70%, with most particles bound by two Fabs and 3D reconstructions confirming bivalent engagement (Figure 2a,c). The Fab form displayed further reduced binding (≈66%), with the majority of particles showing only one Fab per trimer (Figure 2b,c) and ≈16% exhibiting two (Figure 2c; Figure S8, Supporting Information). Notably, full‐length IgG retained bivalent engagement while the third RBD site bound with monovalent Fab was no longer detectable, indicating that avidity allows IgG to compensate for reduced intrinsic affinity. Consistently, Fab exhibited markedly reduced binding and a ≈1,140‐fold drop in neutralization potency (IC_50_ = 3.422 µg/mL; Figure S7b, Supporting Information), while the IgG form maintained high avidity binding and potent neutralization.

To further resolve the precise binding geometry, we determined single particle cryo‐EM structures of the P5‐1C8 IgG bound to SARS‐CoV‐2 WT spike trimers (Figure S9, Supporting Information). We identified two distinct populations of WT S‐P5‐1C8 IgG complexes (Figure 2e). In state 1, the spike trimer adopts a symmetric conformation with all three RBDs in the “up” position. Each trimer accommodates approximately 1.5 IgGs, with one IgG binding two RBDs and the third RBD bound by a separate IgG, potentially bridging adjacent trimers. In state 2, an asymmetric trimer displays two “up” RBDs, both engaged by two Fabs from the same IgG. By docking the crystal structure of RBD‐P5‐1C8 Fab complex (PDB 9K6J) into the EM densities, we measured a distance of 38 Å between Pro217 residues of the heavy chains—well within the ≈65 Å span allowed by the IgG hinge^[^ ^39^, ^53^ ^]^—thereby confirming that the two Fab densities originated from a single IgG molecule and supporting the model of intra‐spike bivalent binding (Figure 2e).

Unexpectedly, P5‐1C8 IgG bound the JN.1 spike in an atypical manner, with particles showing either pronounced trimer aggregation or no detectable IgG‐spike complexes (Figure 2a; Figure S6, Supporting Information). Negative‐stain 2D class averages of well‐dispersed particles confirmed only spike classes without discernible IgG density, indicating the lack of stable complex formation (Figure S10, Supporting Information). To further probe this interaction, we incubated P5‐1C8 IgG with JN.1 spike trimers at an IgG‐to‐spike molar ratio of 3 (≈1.7 µM IgG to ≈0.6 µM spike) for durations ranging from 5 min to 4 h. In all micrographs, dispersed spike trimers showed no detectable evidence of IgG engagement (Figure S11, Supporting Information), suggesting that binding occurred exclusively within aggregated clusters. At ≈28 000‐fold lower IgG concentration (0.06 nM, approximating the IC_50_), dispersed particles remained unbound, with only sparse aggregates observed; further reduction to one‐tenth of the IC_50_ abolished aggregation entirely (Figure S12, Supporting Information). Across all tested conditions, no stable complex between P5‐1C8 IgG and JN.1 spike is captured. The Fab form showed no binding, no aggregation, and no neutralizing activity (IC_50_ > 10 µg/mL, Figure 2b–d; Figure S7c,f, Supporting Information). Unlike the strong binding observed with P5‐1C8 IgG to WT spike, JN.1 spike showed no detectable binding with monovalent Fab, and moderate binding to full‐length IgG (K_D_ = 225 nM) and induced aggregation, suggesting that IgG bivalency engages multiple spike trimers.

### 2.3 Multivalent Binding Mode of Full‐Length P5‑1C8 IgG with NP‐S Nanoparticles

**Figure 3:**
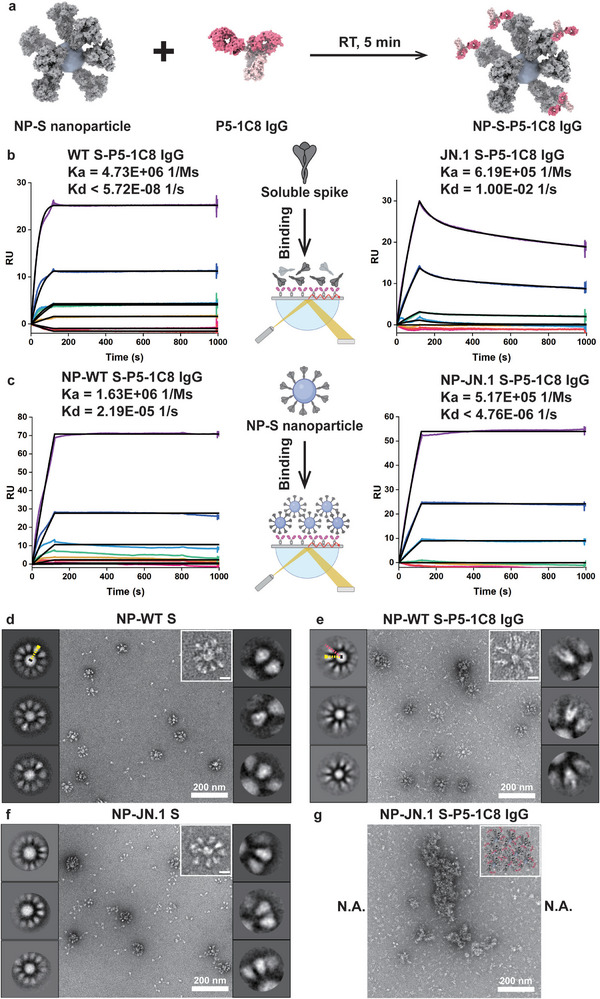
Multivalent binding mode of full‐length P5‑1C8 IgG with NP‐S nanoparticles. a) Schematic illustration of P5‐1C8 IgG binding to NP‐S nanoparticle. b,c) SPR analysis of P5‐1C8 IgG binding to either soluble spike trimers (b) or NP‐S nanoparticles (c) from WT (left) and JN.1 (right). Protein A was immobilized on a CM5 sensor chip to capture P5‐1C8 IgG, and the serial dilutions of either solution spike trimers or NP‐S nanoparticles were flowed through the system. Colored lines represent the raw sensorgrams. Black lines show the best‐fit curves based on experimental data. The calculated association rate (K_a_) and dissociation rate (K_d_) are shown. All results were performed in three independent experiments. The schematic illustrations in the middle panels of (b) and (c) are created with BioRender.com. d,e) Ns‐EM images (middle) and 2D class averages (left and right panels) of NP‐WT S nanoparticles before (d) and after (e) incubation with P5‐1C8 IgG. Scale bar: 200 nm. Inset (upper right) shows zoomed‐in view of a representative nanoparticle. Scale bar: 20 nm. In d (left panel), an auxiliary radius was defined from the NP‐WT S nanoparticle center to the spike tip, yielding ≈30.9 nm. When the same reference radius was applied to e (left panel), the pink line extended ≈7.1 nm beyond it, with this offset corresponding to bound IgG density. f,g) Ns‐EM images (middle) and 2D class averages (left and right panels) of NP‐JN.1 S nanoparticles before (f) and after (g) addition of P5‐1C8 IgG. Scale bar: 200 nm. The upper right insets show a zoomed‐in view of a representative nanoparticle (left) and a schematic illustration of antibody‐induced aggregation (right). The left and right panels of the ns‐EM micrographs in panels (d) to (f) show NP‐S nanoparticles or NP‐S‐IgG complexes and spike or spike‐IgG complexes on nanoparticle, respectively, illustrating representative 2D class averages.

To investigate how full‐length P5‑1C8 IgG engages multiple trimeric spikes, we used nanoparticles (kindly provided by collaborator) coated with spike proteins (NP‐S) to mimic the multivalent display on intact virions (**Figure** **3**a). We first measured this interaction between P5‑1C8 IgG and NP‐S nanoparticles by SPR (Figure 3b,c). To directly compare the binding behavior of soluble spike and NP‐S, P5‐1C8 IgGs were immobilized on Protein A‐coated CM5 sensor chips, followed by injection of either soluble spike (Figure 3b) or NP‐S nanoparticles (Figure 3c). P5‐1C8 IgG exhibited potent binding avidity, with comparable association rates for soluble WT S (4.73 × 10^6^ Ms^−1^) and NP‐WT S (1.63 × 10^6^ Ms^−1^), and extremely slow dissociation rates near the detection limits of the instrument (Figure 3b,c). In contrast, association rates were also similar between soluble and NP‐displayed JN.1 spikes, but their dissociation rates diverged sharply as the soluble form dissociated rapidly (1.00 × 10^−2^ s^−1^; Figure 3b), whereas NP‐presented form exhibited dramatically slower off‐rates (4.76 × 10^−6^ s^−1^; Figure 3c), indicating enhanced stability driven by multivalent interactions. Similarly, cell surface staining assay also analyzes multivalent binding of P5‐1C8 IgG to multiple spikes. By incubating IgG with HEK293T cells expressing various spike proteins from WT to JN.1 SARS‐CoV‐2 variants, revealing comparable binding across all variants with only a modest 1.5‐fold reduction for JN.1 compared to WT (Figure S13, Supporting Information). Compared with the free spike system, systems presenting multiple trimeric spikes, either on nanoparticles or cell surfaces, enable stable binding of full‐length IgG.

We next performed negative‐stain EM to examine the binding mode of P5‐1C8 IgG to NP‐S nanoparticles (Figure 3d‐g). Comparable antibody and spike concentrations were used across NP and free spike systems (≈1.4 µM IgG to ≈0.5 µM spike trimer displayed on nanoparticles). Prior to antibody addition, negative‐stain micrographs and 2D class averages showed uniformly distributed spikes projecting outward from NP surface (Figure 3d,f). We defined an auxiliary radius extending from the NP‐WT S nanoparticle center to the spike tip, yielding ≈30.9 nm (Figure 3d, left panel). After IgG incubation, most nanoparticles remained monodisperse. Applying the same reference radius to NP‐WT S‐P5‐1C8 IgG complexes revealed an additional ≈7.1 nm density beyond the reference, attributable to bound IgG (Figure 3e, left panel). Zoomed‐in views and 2D class averages of selected spikes on nanoparticles further confirmed bivalent binding to NP‐displayed spikes (Figure 3d,e, right panel). Conversely, NP‐JN.1 S exhibited aggregation (Figure 3g). When incubation time was extended to 10 or 30 min, larger and more abundant aggregates were observed in each micrograph (Figure S14, Supporting Information), whereas incubation with monovalent P5‐1C8 Fab resulted in neither binding nor aggregation (Figure S15, Supporting Information), indicating that the bivalency of full‐length IgG is required for cluster formation, similar to the trend seen in free‐solution experiments (Figure S11, Supporting Information). To assess antibody‐induced aggregation under native, virion‐like conditions, Omicron JN.1 pseudoviruses were incubated with P5‐1C8 IgG and analyzed by negative‐stain EM. Aggregation of virus particles was observed (Figure S16, Supporting Information), confirming that IgG promotes JN.1 particle clustering. Overall, enhanced IgG binding was observed with spikes displayed on nanoparticles compared with free spikes, potentially due to multivalent binding to adjacent WT spikes on nanoparticles, while JN.1 revealed only particle aggregation, independent of incubation time, and the underlying process remains unclear.

### 2.4 Molecular Dynamics Simulations Reveal Aggregation Mechanism of P5‐1C8 IgG

**Figure 4:**
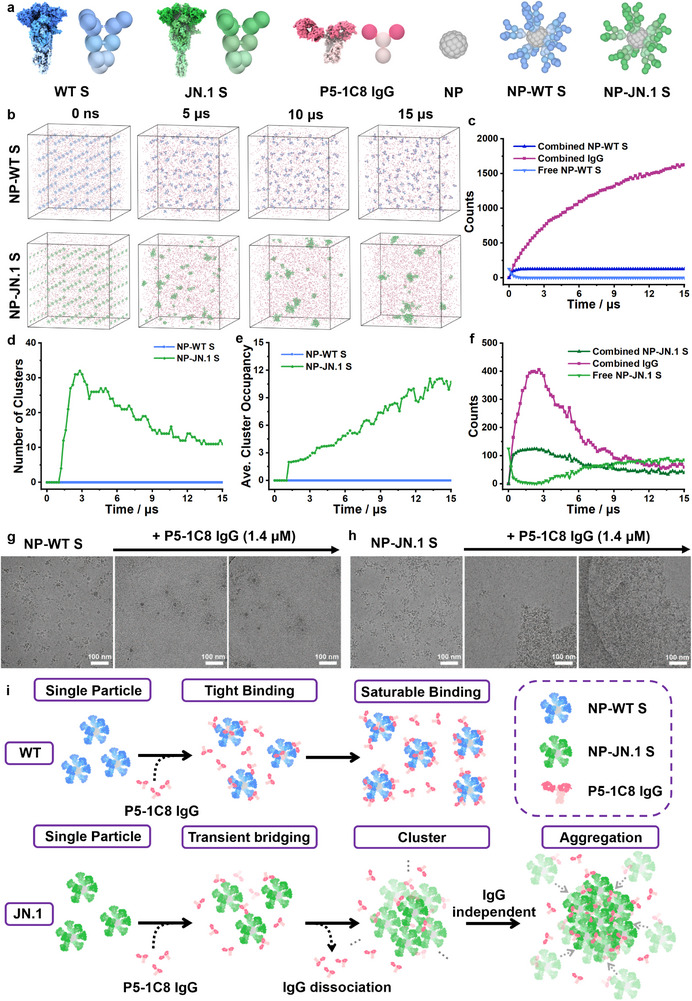
Coarse‐grained molecular dynamics simulations of P5‐1C8 IgG interactions with WT and JN.1 spikes displayed on nanoparticles. a) Coarse‐grained bead representation of spike proteins, IgG molecule, and nanoparticles. WT S is shown in blue, JN.1 S in green, P5‐1C8 IgG in pink, and nanoparticles in grey. b) Representative snapshots of the binding process between P5‐1C8 IgG and either NP‐WT S (top) and NP‐JN.1 S (bottom). Simulations were conducted with 1.4 µM IgG and ≈0.5 µM spike trimer displayed on nanoparticles, within a cubic box of 1500 × 1500 × 1500 nm^3^ containing 125 NP‐S particles, with trajectories were run up to 15 µs. c) Temporal evolution of the number of free or bound P5‐1C8 IgG molecules and NP‐WT S nanoparticles within the simulation box. d,e) Temporal evolution of (d) cluster number and (e) average cluster occupancy in NP‐WT S (blue) or NP‐JN.1 S (green) systems during simulations with P5‐1C8 IgG. f) Temporal evolution of the number of free or bound P5‐1C8 IgG molecules and NP‐JN.1 S nanoparticles within the simulation box. g,h) Representative cryo‐EM images of P5‐1C8 IgG in complex with NP‐WT S (g) or NP‐JN.1 S (h), obtained at the same IgG concentration (1.4 µM), consistent with the simulation conditions. Scale bar: 100 nm. j) Schematic diagram illustrating the binding process and mechanism between P5‐1C8 IgG and either nanoparticle‐presented WT or JN.1spikes.

To investigate how weakly binding P5‐1C8 IgG drives aggregation of JN.1 spike and NP‐JN.1 S, we employed coarse‐grained (CG) molecular dynamics simulations,^[^ ^60^ ^]^ which capture both local binding events and global aggregation. CG models of WT and JN.1 spike trimers and full‐length IgG were constructed to preserve domain architecture and flexibility for bivalent engagement. Specifically, spike trimers were partitioned into four structural segments: residues 1–527 (NTD and RBD), 528–833 (CTD1, CTD2, and FP), 834–1162 (HR1, CH, and CD), and 1163–1240 (HR2 and foldon);^[^ ^61^ ^]^ and full‐length P5‐1C8 IgG was modeled with two Fab fragments, a flexible hinge, and an Fc domain (**Figure** **4**a).^[^ ^62^ ^]^ Simulations were configured with eight spike trimers displayed on 15 nm nanoparticles, as estimated from prior ns‐EM data, and parameterized using experimental KD values and concentrations (1.4 µM IgG and ≈0.5 µM spike trimer). Each simulation box (1500 × 1500 × 1500 nm^3^) contained 125 NP‐S particles, and trajectories were run for up to 15 µs. Upon IgG binding, NP‐WT S remained monodisperse throughout the trajectory (Figure 4b, top; Movie S1, Supporting Information), whereas NP‐JN.1 S exhibited progressive clustering (Figure 4b, bottom; Movie S2, Supporting Information). To quantify these dynamics, we monitored average cluster occupancy, cluster counts, and the populations of free versus bound IgG and nanoparticles (Figure 4c–f). Clusters were defined as assemblies with center‐to‐center separations of less than one nanoparticle diameter. For NP‐WT S, the number of IgG‐bound nanoparticles rapidly plateaued, while the number of nanoparticle‐bound IgG continued to increase, indicating spatially uniform binding across all nanoparticles in the simulation volume and reaching saturation (Figure 4c). This yielded monodisperse NP‐WT S/IgG complexes without detectable clustering (Figure 4d,e). In contrast, NP‐JN.1 S aggregated through a two‐step process. During the initial phase, rapid IgG binding coincided with a synchronous increase in the number of IgG‐bound nanoparticles (Figure 4f) and in cluster counts (Figure 4d). These clusters remained small, with average occupancies below three (Figure 4e), and IgG incorporation peaked at ≈14.2% (Figure 4f). In the second phase, both bound IgG and IgG‐bound nanoparticles declined, while clusters expanded, reflected by fewer total clusters and a steady increase in average occupancy, reaching equilibrium at ≈11 nanoparticles per cluster (Figure 4d–f). Notably, almost all nanoparticles were incorporated into clusters, whereas only ≈2.1% of IgG remained bound (Figure 4f), indicating that IgG primarily drives step‐1 nucleation but is dispensable for step‐2 growth into larger aggregates. Cryo‐EM analysis at the same IgG concentration (1.4 µM) corroborated the simulation results (Figure 4g,h). Prior to IgG addition, NP‐S were uniformly distributed. After IgG incubation, NP‐WT S remained as monodisperse complexes (Figure 4g), while NP‐JN.1 S exhibited amorphous aggregates that predominated across the fields of view (Figure 4h).

Drawing on both MD simulations and experimental data, we generated a mechanistic diagram summarizing the binding and aggregation processes, highlighting the contrasting behaviors of WT and JN.1 spikes upon interaction with P5‐1C8 IgG (Figure 4i). For WT spike, high‐avidity interactions yield stable antibody‐spike complexes that remain anchored on the nanoparticle surface, leading to saturable binding and the formation of stable, monodisperse, single‐nanoparticle assemblies, as consistently observed by ns‐EM, cryo‐EM and MD simulations. In contrast, NP‐JN.1 S undergoes a two‐step process: in step 1, rapid on‐rates drive immediate binding, with multivalent engagement bridging adjacent nanoparticles and nucleating small clusters; in step 2, cluster growth continues, steric crowding displaces a fraction of initially bound IgG, and aggregates expand with minimal IgG remaining engaged. This accelerated, cooperative aggregation mechanism compensates for the reduced monovalent affinity, thereby sustaining potent neutralization despite weakened intrinsic binding.

## 3 Discussion

Binding metrics of Fab or full‐length IgG to monomeric RBD generally correlate with neutralization IC_50_ values,^[^ ^12^, ^13^, ^14^, ^15^ ^]^ but such measurements fail to capture the dynamic conformations states of the native spike trimer^[^ ^19^, ^20^, ^21^ ^]^ and the contribution of full‐length IgG bivalency.^[^ ^63^ ^]^ Notably, certain antibodies deviate from this correlation, and the mechanisms underlying these outliers remain largely unexplored.^[^ ^13^, ^15^, ^42^ ^]^ Herein, antibody P5‐1C8 represents one such exception, highlighting an alternative model of neutralization. All other characterized JN.1‐targeting antibodies show neutralization only when possessing high affinity.^[^ ^64^ ^]^ To dissect its mechanism, we integrated X‐ray crystallography, SPR, negative‐stain and cryo‐EM, and MD simulations to systematically characterize P5‐1C8 IgG binding across spike variants. In both free‐solution and nanoparticle‐displayed systems, P5‐1C8 exhibits sub‐picomolar avidity and potent neutralization against WT spike. MD simulations further revealed rapid and saturable binding, leading to the formation of stable, monodisperse, single‐nanoparticle assemblies. In contrast, while P5‐1C8 binds the JN.1 spike with much lower avidity, it maintains potent neutralizing activity through IgG‐driven aggregation. Simulations demonstrated that small clusters gradually expand into large‐scale aggregates over time. Together, these insights provide a mechanistic framework for “low‐avidity, high‐potency” antibodies and underscore the importance of evaluating multivalent aggregation or cross‐spike bridging during antibody discovery.

Accurately assessing the relationship between affinity, avidity, and neutralization requires careful consideration of experimental design. For example, in SPR assays, different sensor chips, such as CM5, Streptavidin (SA), and NTA, employ distinct immobilization chemistries that can alter protein density, orientation, and conformation, all of which could influence multivalent binding profiles.^[^ ^65^, ^66^ ^]^ In this work, we capturing IgG via Protein A to ensure a consistent Fc‐down orientation, preserving unobstructed access to the Fab regions for antigen binding,^[^ ^65^, ^67^ ^]^ thereby enabling reliable assessment of multivalent interactions with nanoparticle‐displayed spikes. Alternative scaffolds such as DNA origami also hold promise for presenting multiple spikes with precisely programmed geometry. Thus, selecting an appropriate immobilization strategy is essential for accurately evaluating antigen‐antibody multivalency, particularly for antibodies like P5‐1C8, whose potency depends on multivalent stabilization rather than high‐affinity monovalent binding.

Structural analyses further reveal that P5‐1C8 neutralizes SARS‐CoV‐2 through a distinct mechanism of multivalent aggregation. A similar case is observed for the SARS‐CoV‐2 antibody P5‐1H1, which shows undetectable binding yet high neutralization.^[^ ^42^ ^]^ P5‐1H1 targets highly overlapping epitopes with a comparable approach angle (Figure S17a, Supporting Information),^[^ ^32^ ^]^ and displays a similar time‐dependent aggregation pattern (Figure S17b, Supporting Information). More cases of aggregation‐driven neutralization by antiviral antibodies have been reported—for example, influenza antibody HC19,^[^ ^5^ ^]^ Chikungunya virus antibody CHK‐124,^[^ ^4^ ^]^ and SARS‐CoV‐2 antibody W328‐6H2,^[^ ^27^ ^]^—but those cases involve inherently high affinity and were visualized only as endpoint states by EM. EM captures static snapshots, and despite varying our incubation times down to 5 minutes, we failed to observe intermediate assemblies that could illuminate the 3D binding mode. To bridge that gap, we applied molecular dynamics simulations informed by our own experimental K_D_, structural data, and concentration. In contrast to high‐affinity antibodies, whose aggregation is driven by direct antigen‐antibody‐antigen crosslinking, low‐affinity antibodies operate via a distinct mechanism: rapid on‐rates enable transient inter‐spike bridging, and once initial clusters are nucleated, their growth proceeds largely independently of continued IgG engagement, potentially driven by weak spike‐spike interactions that are reinforced when multivalently displayed spikes are brought into close proximity. To our knowledge, this study is the first to dissect the dynamics of low‐affinity antibody‐mediated aggregation, and we hope it sparks more systematic theoretical and experimental evaluation of multivalent synergy in weakly binding antibodies, enriching both mechanistic understanding and future therapeutic discovery.

## 4 Experimental Section

### Cell Lines

HEK293T cells (ATCC, CRL‐3216; RRID: CVCL_0063) were cultured at 37 °C with 5% CO_2_ in Dulbecco's minimal essential medium (DMEM) supplemented with 10% (v/v) heat‐inactivated fetal bovine serum (FBS) and 100 U/mL penicillin‐streptomycin. FreeStyle 293F cells (Thermo Fisher Scientific, R79007; RRID: CVCL_D603) were maintained at 37 °C in 5% CO_2_ in SMM 293‐TII expression medium (Sino Biological, M293TII). The cell lines were obtained from commercial suppliers and handled under standard aseptic conditions. Throughout the study expected morphology and consistent reproducible protein expression across independent replicates and batches were observed, with no signs of contamination.

### Protein Expression and Purification

Codon‐optimized genes encoding trimeric spike proteins of SARS‐CoV‐2 WT, BA.1, BA.5, and JN.1 were synthesized by Tsingke Co., Ltd. and cloned into the SARS‐CoV‐2 S HexaPro vector (Addgene: 154754). Each construct encoded residues 1–1208 of spike protein, incorporating six proline substitutions (F817P, A892P, A899P, A942P, K986P, V987P), a “GSAS” substitution at the furin cleavage site (residues 682–685) to enhance stability, and a C‐terminal T4 fibritin (folden) trimerization motif, an HRV3C protease cleavage site, 8×HisTag and Twin‐Strep Tag. Proteins were expressed in FreeStyle 293F cells and harvested 96‐h post‐transfection. Supernatants were clarified and purified by Strep‐Tactin Sepharose (YEASEN: #20495ES25), followed by size‐exclusion chromatography using a Superose 6 Increase 10/300 GL column (Cytiva: #29091596) in TBS buffer. Purity and molecular weight were assessed by SDS‐polyacrylamide gel electrophoresis (SDS‐PAGE).

For the receptor‐binding domain (RBD) of the SARS‐CoV‐2 prototype (residues Arg319‐Lys529), expression was similarly carried out in FreeStyle 293F cells. The RBD construct included a C‐terminal 8×His tag and was purified using Ni‐NTA affinity chromatography followed by gel filtration in PBS. Protein purity was confirmed by SDS‐PAGE. Detailed characterization of all protein constructs is provided in Table S2 (Supporting Information).

### Antibody and Fab Fragment Production

Antibody P5‐1C8 is a potent neutralizing mAb initially isolated from COVID‐19 convalescents following ancestral SARS‐CoV‐2 infection.^[^ ^32^ ^]^ The antibody is covered by patent protection (Patent NO. 2023100460521). The variable regions of its heavy and light chains were cloned into expression vectors containing the human IgG1 constant regions, as previously described. Briefly, plasmids encoding the heavy and light chains were co‐transfected into HEK293 F cells using polyethylenimine (PEI, Yeasen). After 96 hours, culture supernatants were harvested, and antibodies were purified using AmMag^TM^ Protein A Magnetic beads (GenScript). Bound antibodies were eluted with glycine buffer (pH 2.0) and further purified by size‐exclusion chromatography on a Superdex 200 Increase 10/300 GL column (Cytiva: #28990944).

Fab fragments were generated by papain digestion (Beijing Lablead: #P3250) using an IgG‐to‐papain ratio of 25:1 (w/w) in digestion buffer (100 mM Tris, 2 mM EDTA, 10 mM L‐cysteine) at 37 °C for approximately 5 hours. Fc fragments were removed using Protein A Magnetic beads. The concentrations of IgG and Fab were determined by a Nanodrop 600Plus Spectrophotometer (JIAPENG, Shanghai), and final purification was performed by gel filtration using the same Superdex 200 column. Detailed characterization of the IgG and Fab is summarized in Table S2 (Supporting Information).

### Production of Pseudoviruses and Neutralization Assay

Pseudoviruses were generated using the wild‐type SARS‐CoV‐2 (GenBank: MN908947.3) and several variants. The Beta variant (Pango lineage B.1.351, GISAID: EPI_ISL_700450) carried 10 spike mutations: L18F, D80A, D215G, 242 244del, S305T, K417N, E484K, N501Y, D614G, and A701V. The Delta variant (Pango lineage B.1.617.2, GISAID: EPI_ISL_1534938) included mutations T19R, G142D, 156–157del, R158G, A222V, L452R, T478K, D614G, P681R, D950N. The BA.1 variant (Pango lineage BA.1, GISAID: EPI_ISL_6752027) contained 32 spike mutations, including A67V, Δ69‐70, T95I, G142D/Δ143‐145, Δ211/L212I, ins214EPE, G339D, S371L, S373P, S375F, K417N, N440K, G446S, S477N, T478K, E484A, Q493R, G496S, Q498R, N501Y, Y505H, T547K, D614G, H655Y, N679K, P681H, N764K, D796Y, N856K, Q954H, N969K, and L981F. The BA.2.75 spike construct was based on BA.2 with additional mutations W152R, F157L, I210V, G257S, D339H, G446S, N460K, and Q493R (reverted). The BA.5 variants (Pango lineage BA.5, GISAID: EPI_ISL_12559461) was constructed with 30 mutations in the spike such as T19I, 24–26del, A27S, G142D, V213G, G339D, S371F, S373P, S375F, T376A, D405N, R408S, K417N, N440K, L452Q, S477N, T478K, E484A, F486V, Q498R, N501Y, Y505H, D614G, H655Y, N679K, P681H, N764K, D796Y, N969K and Q954H. BF.7 (Pango lineage BF.7, GISAID: EPI_ISL_15429967), BQ.1 (Pango lineage BQ.1, GISAID: EPI_ISL_15458271), BQ.1.1 (Pango lineage BQ.1.1, GISAID: EPI_ISL_15458263) were derived from the BA.5 background and included additional of R346T, K444T, and N460K, or all of three mutation sites. The XBB variant (Pango lineage XBB, GISAID: EPI_ISL_15601178) featured 13 mutation sites of V83A, Δ145, H146Q, Q183E, V213E, G339H, R346T, L368I, V445P, G446S, N460K, F486S, F490S, and the removal of Q493R, compared to BA.2 variant. Addition of G252V to XBB resulted in XBB.1 (PANGO lineage XBB.1, GISAID: EPI_ISL_15596825). The Omicron JN.1 variant (Pango lineage JN.1, GISAID: EPI_ISL_19019427) was constructed with 61 mutations in the spike including T19I, R21T, L24del, P25del, P26del, A27S, S50L, H69del, V70del, V127F, G142D, Y144del, F157S, R158G, N211del, L212I, V213G, L216F, H245N, A264D, I332V, G339H, K356T, S371F, S373P, S375F, T376A, R403K, D405N, R408S, K417N, N440K, V445H, G446S, N450D, L452W, L455S, N460K, S477N, T478K, N481K, V483del, E484K, F486P, Q498R, N501Y, Y505H, E554K, A570V, D614G, P621S, H655Y, I670V, N679K, P681R, N764K, D796Y, S939F, Q954H, N969K. P1143L.

Pseudoviruses were produced by co‐transfecting HEK‐293T cells (ATCC) with human immunodeficiency virus backbones expressing firefly luciferase (pNL4‐3‐R‐E‐luciferase) and pcDNA3.1 vectors encoding the SARS‐CoV‐2 spike protein variants. Viral supernatants were harvested 48–72 h post‐transfection, clarified by centrifugation, and stored at −80 °C until use.

Neutralization assays were performed by incubating serial dilutions of antibodies with pseudoviruses at 37 °C for 1 h. The mixture was then added to 293T‐hACE2 cells. After 48 h, cells were lysed, and luciferase activity was measured. The percentage of neutralization was calculated relative to virus‐only control. Antibody dilutions for P5‐1C8 IgG or Fab started at 10 µg/mL and were tested against pseudoviruses bearing the spike proteins of SARS‐CoV‐2 WT, Beta, Delta, BA.1, BA.2.75, BA.5, BF.7, BQ.1, BQ.1.1, XBB, XBB.1, and JN.1. The half‐maximal inhibitory concentration (IC_50_) was calculated using a four‐parameter dose inhibition response model in Graphpad Prism 8.0.

### Crystallization and Data Collection

Fab fragments were mixed with SARS‐CoV‐2 WT RBD at a molar ratio of 1:2 and incubated overnight at 4 °C. The resulting complexes were further purified by gel‐filtration chromatography and concentrated to 10 mg mL^−1^ in TBS buffer for crystallization. Initial crystallization screening was performed at 18 °C in 96‐well plates using the micro‐volume sitting‐drop vapor diffusion method, with crystallization kits listed in Table S5 (Supporting Information). A Mosquito liquid handling robot (TTP Labtech) was used to mix 200 nL of protein solution with 200 nL of reservoir solution. Crystals were initially observed in conditions containing 0.2 M ammonium phosphate dibasic, 20% (w/v) polyethylene glycol (PEG) 3,350, pH 8.0 (PEG/ION NO.44). Subsequent optimization was conducted using the sitting‐drop vapor diffusion method in 24‐well plates. In this stage, 1 µL of protein solution was mixed with 1 µL of reservoir solution. High‐quality crystals of the RBD‐Fab complex were obtained in 0.2 M ammonium phosphate dibasic, 28% (w/v) PEG 3,350, pH 8.0. The workflow for crystal optimization procedure is illustrated in Figure S2 (Supporting Information). X‐ray diffraction data were collected at the BL02U1 beamline of the Shanghai Synchrotron Research Facility (SSRF) and auto‐processed with the HKL3000 software. Data collection and processing statistics are summarized in Table S7 (Supporting Information).

### Structure Determination and Refinement

The structure of the SARS‐CoV‐2 WT RBD‐P5‐1C8 Fab complex was solved by molecular replacement using PHENIX.^[^ ^68^ ^]^ Model building and iterative refinement were performed with COOT^[^ ^69^ ^]^ and PHENIX,^[^ ^68^ ^]^ respectively. Final Ramachandran statistics: 96.98% favored, 3.02% allowed, and 0.00% outliers for the final model. Refinement statistics are listed in Table S7 (Supporting Information). All structure figures were generated using UCSF ChimeraX.^[^ ^70^ ^]^

### Negative‐Stain Electron Microscopy

Grid preparation, imaging, and data processing were carried out following established protocols with minor modifications.^[^ ^71^ ^]^ Briefly, SARS‐CoV‐2 spike proteins (WT, BA.1, BA.5, or JN.1) were incubated with P5‐1C8 IgG or Fab, and P5‐1H1 IgG (for BA.5 spike only), at a 3:1 Fab‐to‐protomer molar ratio at room temperature for 0.5‐4 hours. The final concentration of the complexes was approximately ≈0.3 µg/µL. Prior to grid application, samples were diluted to 0.015 µg/mL in 1× TBS buffer. A 3.5 µL aliquot was applied to 200‐mesh copper grids (Zhongjingkeyi Films Technology Co., Ltd, Beijing), blotted with filter paper, and stained with 2% (w/v) uranyl acetate. Micrographs were acquired using a JEOL JEM‐2100F high‐resolution transmission electron microscope operating at 200 kV, with images collected at 30 000× magnification using SerialEM automated software. Image processing and 2D/3D reconstruction were performed using Relion 4.0.1,^[^ ^72^ ^]^ and visualization and alignment of the final EM maps were carried out in UCSF Chimera and ChimeraX.^[^ ^70^ ^]^ Negative‐stain EM maps have been deposited in the Electron Microscopy Data Bank (EMDB; www.emdataresource.org), with accession codes provided in Table S3 (Supporting Information).

### Cryo‐EM Sample Preparation and Data Acquisition

Purified SARS‐CoV‐2 WT spike protein was mixed with P5‐1C8 IgG at a 3:1 molar ratio of IgG to spike trimer and incubated at room temperature for 2 h. The final concentrations of the complex were adjusted to 1 mg/mL in TBS buffer. Prior to vitrification, Quantifoil R1.2/1.3 Cu 300‐mesh grids were glow‐discharged for 20 s at medium power using a plasma cleaner. A 4 µL aliquot of the complexes was applied to the grids, using a blot force of ‐2 and a 3‐second blot time at 100% relative humidity and 4 °C, and plunge‐frozen in liquid ethane using FEI Vitrobot system. Cryo‐EM data were acquired on a FEI Titan Krios transmission electron microscope (Thermo Fisher Scientific) operating at 300 kV, equipped with a Gatan K3 Summit direct electron detector. Automated data collection was performed using SerialEM software at 29 000x magnification, corresponding to a pixel size of 0.97 Å. Images were collected at a defocus range of −1.0–−1.6 µm. Each movie stack was recorded with a total electron dose of 50 e/Å^2^ fractionated in 32 frames.

### Cryo‐EM Data Processing

Cryo‐EM micrographs were processed using CryoSPARC v4.6.0.^[^ ^73^ ^]^ Contrast transfer function (CTF) parameters were estimated using the Patch CTF Estimation. Initial particle picking was performed with Blob Picker, followed by reference‐free 2D classification. Based on these results, the Template Picker was applied to re‐pick particles, which were then subjected to a second round of 2D classification. Particles were extracted using a 480‐pixel box size and refined through several iterative rounds of 2D classification. Well‐defined particles were used for Ab‐Initio Reconstruction and Homogeneous Refinement. The resulting dataset was then imported into Relion for 3D classification to eliminate low‐quality and heterogeneous particles. Composite masks targeting RBD‐Fab and full Spike‐Fabs regions were applied during classification to resolve distinct conformational states, including particles with two RBDs in the “up” position and one “down”, or all three RBDs in the “up” position. Subsequently, particles from selected classes were re‐imported into CryoSPARC for Local refinement. Final cryo‐EM maps were interpreted by fitting atomic models using UCSF Chimera.^[^ ^70^ ^]^ Both the RBD and the P5‐1C8 Fab structures were derived from crystallographic data (PDB 9K6J; https://www.rcsb.org/). All cryo‐EM visualizations were rendered in Chimera or ChimeraX. Data collection parameters and the complete processing workflow are summarized in Table S6 (Supporting Information) and illustrated in Figure S9 (Supporting Information), respectively.

### Binding of P5‐1C8 Antibody to Cell Surface‐Expressed Spike Glycoproteins

HEK293T cells were transfected with plasmids encoding full‐length spike proteins of various SARS‐CoV‐2 variants and incubated at 37 °C for 36 h. Following incubation, cells were detached using trypsin and seeded into 96‐well plates. Between each staining step, cells were washed twice with 200 µL of staining buffer (PBS containing 2% heated‐inactivated fetal bovine serum). To assess cross‐reactive binding, cells were first incubated with the P5‐1C8 antibody at a concentration of 5 µg/mL in 100 µL staining buffer for 30 minutes at 4 °C. The tested spike variants included SARS‐CoV‐2 WT, Beta, Delta, BA.1, BA.2.75, BA.5, BF.7, BQ.1, BQ.1.1, XBB, XBB.1, and JN.1. After washing, cells were stained with PE‐conjugated anti‐human IgG (H+L) (Biolegend 410708) in 50 µL staining buffer for 30 min at 4 °C. Following additional washes, cells were resuspended and analyzed using a BD LSRFortessa flow cytometer (BD Biosciences, USA). Data were processed with FlowJo v10 (FlowJo, USA). Mock‐transfected cells served as negative controls. MFI (Mean Fluorescence Intensity) was calculated and exported by FlowJo.

### Surface Plasmon Resonance (SPR) Analysis of IgG and Fab Binding to SARS‐CoV‐2 Spike Trimer

Binding kinetics and affinity of P5‐1C8 IgG or Fab fragments to SARS‐CoV‐2 spike trimers (WT, BA.1, and JN.1) were measured using a Biacore 8K SPR system (Cytiva). His‐tagged spike trimers were immobilized on a Ni^2+^‐charged NTA sensor chip, followed by injection of P5‐1C8 IgG or Fab as analytes. NTA surfaces were regenerated with 350 mM EDTA, recharged with 0.5 mM NiCl_2_, and activated with EDC/NHS. Final immobilization levels were ≈2900 RU (WT), ≈4900 RU (BA.1), and ≈6000 RU (JN.1). Binding assays were conducted at a flow rate of 30 µL/min in PBST buffer. Serial dilutions of P5‐1C8 IgG or Fab were injected over the immobilized spike protein for 120 s, followed by a dissociation phase of 900 s (WT) or 360 s (BA.1 and JN.1), using a multi‐cycle method. The sensor surface was regenerated between cycles with 10 mM glycine‐HCl (pH 2.0) for 60 s at a flow rate of 30 µL/min. Kinetic parameters were determined by fitting the reference‐subtracted sensorgrams to a 1:1 (Langmuir) binding model (WT and BA.1) or bivalent analyte model (JN.1) using Biacore Insight Evaluation Software (Cytiva).

### Ns‐EM Analysis of Antibody Interacting with Spike Displayed on Nanoparticle

NP‐S nanoparticles used in this study were supplied by the collaborator, and the formulation procedure, based on a confidentiality agreement, cannot be disclosed in detail. These NP‐S nanoparticles were further incubated with P5‐1C8 IgG at a 3:1 IgG‐to‐Spike molar ratio for 5–30 min at room temperature, maintaining a final concentration of ≈0.3 µg/µL. Prior to grid preparation, samples were diluted to 0.015 µg/µL in 1× TBS buffer. A 3.5 µL aliquot of the diluted sample was applied to 200‐mesh copper grids (Zhongjingkeyi Films Technology Co., Ltd), blotted with filter paper, and stained with 2% (w/v) uranyl acetate. Imaging the nanoparticle states and complexes was performed using a transmission electron microscope operated at 80 kV.

### Cryo‐EM Analysis of Antibody Interacting with Spike Displayed on Nanoparticle

Following a similar procedure to the negative‐stain protocol, these NP‐S nanoparticles were incubated with P5‐1C8 IgG at varying IgG‐to‐Spike molar ratios for 5 min at room temperature, maintaining a final concentration of ≈0.3 µg/µL. For cryo‐EM grid preparation, Quantifoil R1.2/1.3 Cu 300‐mesh grids were glow‐discharged for 20 s at medium power using a plasma cleaner. A 4 µL aliquot of the sample was applied to each grid, blotted with a force setting of ‐2 and a 3‐second blot time at 100% relative humidity and 4 °C, and plunge‐frozen in liquid ethane using a FEI Vitrobot system. Cryo‐EM images were acquired on a Thermo Fisher Talos transmission electron microscope operating at 200 kV, using a nominal magnification of 92 000x, which corresponds to a pixel size of 1.59 Å.

### SPR Analysis of IgG Binding to Soluble and Nanoparticle‐Displayed Spike Proteins

SPR assays were conducted using a Biacore 8K system (Cytiva) to evaluate the binding kinetics between P5‐1C8 IgG and either soluble spike trimers or nanoparticle‐displayed spike (NP‐S) constructs. Protein A was covalently immobilized onto the CM5 chip via standard amine coupling in 10 mM sodium acetate buffer (pH 4.0), and P5‐1C8 IgG was subsequently captured on the Protein A surface. SPR measurements were carried out at a flow rate of 30 µL/min in PBST buffer (PBS with 0.05% Tween‐20). Serial dilutions of either soluble spike or NP‐S nanoparticles were injected over the sensor surface for 120 s, followed by a dissociation phase of 900 s. The chip surface was regenerated between cycles using 10 mM glycine‐HCl (pH 2.0) for 60 s. Sensorgrams were reference‐subtracted and analyzed using the Biacore Insight Evaluation Software (Cytiva), and kinetic parameters were obtained by fitting to a 1:1 (Langmuir) binding or bivalent analyte model.

### Negative‐Stain EM Analysis of Antibody‐Induced Virus Aggregation

Concentrated SARS‐CoV‐2 JN.1 pseudovirus (Genomeditech Co., Ltd, Shanghai) were purified by size‐exclusion chromatography on a Superose 6 Increase 10/300 GL column (Cytiva, #29091596) equilibrated in PBS. Purified JN.1 pseudovirus was incubated with P5‐1C8 IgG for 1 h at room temperature. Subsequently, A 3.5 µL aliquot was applied to 200‐mesh copper grids, adsorbed for 5 min, blotted, and stained with 2% (w/v) uranyl acetate, then air‐dried. Grids were examined by transmission electron microscope operated at 80 kV, and multiple, randomly selected fields were imaged to assess antibody‐induced aggregation. All procedures were conducted under appropriate biosafety conditions.

### Coarse‐Grained Methods, Molecular Dynamics, and Potential Energy Settings

Coarse‐grained (CG) molecular dynamics (MD) simulations^[^ ^60^, ^74^, ^75^ ^]^ were employed to examine the binding and aggregation behavior of P5‐1C8 IgG interacting with either SARS‐CoV‐2 WT or JN.1 spike trimers displayed on nanoparticles. The CG methodology simplifies the molecular system by representing groups of atoms as single interaction sites, or “beads”, reducing computational complexity while preserving key structural and dynamic features. In this model, each residue of spike and antibody structures was mapped onto one or more CG beads based on its chemical properties, with hydrophobic, polar, and charged side chains grouped accordingly, and the backbone represented by separate beads. The length unit σ was set to 7.5 nm, the time unit t_0_ to 0.1 us, and the energy unit ε to k_B_T at 300 K, where k_B_ is the Boltzmann constant (k_B_ =  1.38  ×  10^−23^ J K^−1^):

(1) $$\varepsilon =k_{B}\;T=1.38\;\times 10^{-23}J\;K^{-1}\;\times 300K=4.14\;\times 10^{-21}J$$

The molecular dimensions were defined as follows: the nanoparticle radius (R_NP_) was set to 7.5 nm, spike trimer length (L_Spike_) was 25 nm, and antibody length (L_IgG_) was 15 nm. The molecular masses were assigned as M_Spike_ =  540 kg mol^−1^ for WT or JN.1 spike protein and M_IgG_ =  150 kg mol^−1^ for antibody.

CG‐MD simulations were performed using the GALAMOST software package^[^ ^76^ ^]^ within the *NVT* ensemble to maintain a constant temperature of 300 K. Temperature control was achieved through a velocity rescaling thermostat with a coupling constant of 0.2 ps, while pressure was regulated using Nose–Hoover thermostat with a coupling constant of 1 ps and a thermostat coupling parameter of 0.5. For simulations involving spike trimers displayed on nanoparticles, a cubic box with fixed dimensions of 1500×1500×1500 nm^3^ was used to accommodate a large number of nanoparticles. To better simulate experimental conditions, the system included ≈0.5 µM spike trimer displayed on nanoparticles and 1.4 µM IgG, with parameters and methodologies ensuring representation of the essential system physics. The equations of motion were integrated using the Velocity‐Verlet algorithm with a time step of 4 ns. Each simulation was run for a total duration of 15 µs, a timescale sufficient to capture both short‐term binding events and long‐term aggregation processes. Trajectories were saved at 1 ns interval for subsequent analysis.

Interactions between CG beads were described using a combination of bonded and non‐bonded potentials. Bonded potentials included harmonic terms for bond lengths and angles, as well as dihedral potentials to maintain the overall molecular structure. The bond length potential was typically modeled using a harmonic potential, which can be expressed as:

(2) $$U_{\mathrm{bond}}\;\left(r\right)=\frac{1}{2}\;k_{b}{\left(r-r_{0}\right)}^{2}$$

where U_bond_(r) represents the potential energy as a function of bond length, *k*_b_ is the force constant that determines the bond stiffness, and r_0_ is the equilibrium bond length. This harmonic potential ensures that the distance between two bonded beads remains close to the equilibrium value r_0_, with deviations leading to an increase in potential energy. The bond angle potential is typically modeled using a harmonic potential:

(3) $$U_{\mathrm{angle}}\;\left(\theta \right)=\frac{1}{2}\;k_{\theta }{\left(\theta -\theta_{0}\right)}^{2}$$

where U_angle_(θ) is the potential energy as a function of bond angle θ, k_θ_ is the force constant for angle, and θ_0_ is the equilibrium bond angle. This potential maintains the preferred angle between three consecutive beads, thereby contributing to the overall conformational stability of the molecule.

Non‐bonded potentials describe the interactions between beads that are not directly connected, including van der Waals (VDW) and electrostatic interactions. The Lennard–Jones (LJ) potential is commonly used to model van der Waals interactions:

(4) $$U_{\mathrm{LJ}}\left(r\right)=4\varepsilon \left[{\left(\frac{\sigma }{r}\right)}^{12}-{\left(\frac{\sigma }{r}\right)}^{6}\right]$$

where U_LJ_(r) is the potential energy as a function of the distance *r* between two CG beads, ε is the potential well depth representing the interaction strength, and σ is the distance at which the potential energy is zero, related to the size of the interacting beads. The LJ potential captures both the attractive and repulsive forces between non‐bonded beads, providing a realistic description of van der Waals interactions. Electrostatic interactions are typically modeled using the Coulomb potential:

(5) $$U_{\mathrm{Coulomb}}\;\left(r\right)=\frac{q_{i}q_{j}}{4\pi \varepsilon_{0}\varepsilon_{r}r}\;$$

where U_Coulomb_(r) is the potential energy as a function of the distance *r* between two charged beads, ε_0_is the permittivity of free space, and ε_*r*_is the relative permittivity (dielectric constant) of the medium. In this study, non‐bonded interactions were modeled using Lennard–Jones potential for van der Waals forces and Coulomb potential for electrostatic interactions, parameterized based on the MARTINI force field. Electrostatic interactions were treated using the reaction field method to account for the screening effect of solvent. To clarify, the full simulation parameters, along with their values and sources, are summarized in Table S8 (Supporting Information).

### Analysis of the CG‐MD Simulations

All analyses were carried out by means of house‐made python scripts employing the Scipy,^[^ ^77^ ^]^ Pytorch,^[^ ^78^ ^]^ Scikit‐learn,^[^ ^79^ ^]^ and Fresnel (https://fresnel.readthedocs.io/) package. Snapshots in Figure 4b and Movies S1 and S2 were rendered with Fresnel package using GPU acceleration. For the binding analysis of nanoparticle and antibody, proceed as follows: two spikes and antibody were considered in contact (i.e., they belonged to the same assembly) if any of their CG beads lied within a distance of r_cut_=  s  =  7.5 nm. A nanoparticle was considered to be bound if at least one of its grafted spikes contacted an antibody. For aggregation analysis, cluster identification was performed using the DBSCAN algorithm in SciPy. The epsilon parameter, defining the maximum neighbor distance between nanoparticles, was set to 30.0 nm, and the minimum cluster occupancy of 2 was specified to classify even the smallest aggregates while designating isolated points as noise. This density‐based approach effectively identifies clusters of arbitrary shapes within simulation trajectories, providing key insights into oligomerization and aggregation processes in simulation.

### Quantification and Statistical Analysis

Protein concentrations were determined using a Nanodrop spectrophotometer. SDS‐PAGE gels, size‐exclusion chromatography profiles, and negative‐stain EM micrographs of purified IgG, Fab, spike proteins, and RBD are summarized in Table S2 (Supporting Information). Neutralization assays were performed in two independent experiments, each with duplicate measurements. Half‐maximal inhibitory concentration (IC_50_) were calculated by the equation of four‐parameter dose inhibition response using Graphpad Prism 8.0. SPR binding kinetics were conducted in triplicate, and sensorgrams were reference‐subtracted and fitted using appropriate kinetic models using Biacore Insight Evaluation 5.0.18.22102. For ns‐EM and cryo‐EM analyses, the number of micrographs, initial and final particle counts, and class distributions are summarized in Tables S3 and S6 (Supporting Information). Data processing and visualization were carried out using CryoSPARC v4.6.0, Relion v4.0.1, Phenix, Coot, UCSF Chimera, UCSF ChimeraX, Biacore Insight Evaluation 5.0.18.22102, Origin 2018, GraphPad Prism v8.0, FlowJo v10, and custom Python scripts employing SciPy, PyTorch, Scikit‐learn, and Fresnel libraries. Coarse‐grained MD simulations were performed with the GALAMOST package. EM density maps and crystallographic models have been deposited in EMDB and PDB.

## Conflict of Interest

The authors declare no conflict of interest.

## Author Contributions

N.L., P.C., and X.D. contributed equally to this work. Y.Y., Y.Z., L.Z., Q.Z., L.Y., and Z.L. conceived and designed the study. N.L., P.C., and X.D. performed most of the experiments, with assistance from H.X., Z.L., Z.S., J.L., and F.G. N.L. performed biophysical and structural assays, and solved and analyzed the ns‐EM and cryo‐EM structures of the antibody‐spike complex. N.L. solved the crystal structure of WT RBD‐P5‐1C8 Fab complex with assistance from Z.L., Z.S., F.G., S.F., and Y.Y. Q.Z., P.C., and J.L. performed functional assays, including flow cytometry and pseudovirus neutralization assays. N.L. and P.C. concentrated and purified the SARS‐CoV‐2 JN.1 pseudoviruses. X.D. and L.Y. performed the molecular dynamics simulations. Y.C., J.L., Y.H., G.D., Y.J., L.C., X.N., H.Z., K.Z., Y.D., D.L., X.W., D.H., X.P., and C.C. provided additional technical or intellectual assistance. N.L., P.C., X.D., Z.L., L.Y., Q.Z., L.Z., Y.Z., and Y.Y. had full access to data in the study, generated figures and tables, and took responsibility for the integrity and accuracy of the data presentation. Y.Y. and N.L. wrote the manuscript. All authors reviewed and approved the final version of the manuscript.

## Supporting information



Supporting Information

Supplemental Movie 1

Supplemental Movie 2

## Data Availability

The data that support the findings of this study are available from the corresponding author upon reasonable request.

## References

[1] H. Qi , B. Liu , X. Wang , L. Zhang , Nat. Immunol. 2022, 23, 1008. 35761083 10.1038/s41590-022-01248-5

[2] Y. Chen , X. Zhao , H. Zhou , H. Zhu , S. Jiang , P. Wang , Nat. Rev. Immunol. 2023, 23, 189. 36168054 10.1038/s41577-022-00784-3 PMC9514166

[3] R. Yan , R. Wang , B. Ju , J. Yu , Y. Zhang , N. Liu , J. Wang , Q. Zhang , P. Chen , B. Zhou , Y. Li , Y. Shen , S. Zhang , L. Tian , Y. Guo , L. Xia , X. Zhong , L. Cheng , X. Ge , J. Zhao , H. W. Wang , X. Wang , Z. Zhang , L. Zhang , Q. Zhou , Cell Res. 2021, 31, 517. 33731853 10.1038/s41422-021-00487-9 PMC7966918

[4] Q. F. Zhou , J. M. Fox , J. T. Earnest , T. S. Ng , A. S. Kim , G. Fibriansah , V. A. Kostyuchenko , J. Shi , B. Shu , M. S. Diamond , S. M. Lok , Proc. Natl. Acad. Sci. USA 2020, 117, 27637; 33087569 10.1073/pnas.2008051117 PMC7959576

[5] J. A. Williams , L. Gui , N. Hom , A. Mileant , K. K. Lee , J. Virol. 2018, 92, 02006. 10.1128/JVI.02006-17 PMC5827376 29263254

[6] D. Pinto , M. M. Sauer , N. Czudnochowski , J. S. Low , M. A. Tortorici , M. P. Housley , J. Noack , A. C. Walls , J. E. Bowen , B. Guarino , L. E. Rosen , J. di Iulio , J. Jerak , H. Kaiser , S. Islam , S. Jaconi , N. Sprugasci , K. Culap , R. Abdelnabi , C. Foo , L. Coelmont , I. Bartha , S. Bianchi , C. Silacci‐Fregni , J. Bassi , R. Marzi , E. Vetti , A. Cassotta , A. Ceschi , P. Ferrari , et al., Science 2021, 373, 1109. 34344823 10.1126/science.abj3321 PMC9268357

[7] B. S. Thompson , B. Moesker , J. M. Smit , J. Wilschut , M. S. Diamond , D. H. Fremont , PLoS Pathog. 2009, 5, 1000453; 10.1371/journal.ppat.1000453 PMC2679195 19478866

[8] C. D. Murin , I. A. Wilson , A. B. Ward , Nat. Microbiol. 2019, 4, 734. 30886356 10.1038/s41564-019-0392-y PMC6818971

[9] J. Jin , N. M. Liss , D. H. Chen , M. Liao , J. M. Fox , R. M. Shimak , R. H. Fong , D. Chafets , S. Bakkour , S. Keating , M. E. Fomin , M. O. Muench , M. B. Sherman , B. J. Doranz , M. S. Diamond , G. Simmons , Cell Rep. 2015, 13, 2553. 26686638 10.1016/j.celrep.2015.11.043 PMC4720387

[10] D. R. Burton , Nat. Rev. Immunol. 2023, 23, 720; 37069260 10.1038/s41577-023-00858-w PMC10108814

[11] J. Dufloo , T. Bruel , O. Schwartz , Retrovirology 2018, 15, 51. 30055632 10.1186/s12977-018-0434-1 PMC6064125

[12] Y. Cao , B. Su , X. Guo , W. Sun , Y. Deng , L. Bao , Q. Zhu , X. Zhang , Y. Zheng , C. Geng , X. Chai , R. He , X. Li , Q. Lv , H. Zhu , W. Deng , Y. Xu , Y. Wang , L. Qiao , Y. Tan , L. Song , G. Wang , X. Du , N. Gao , J. Liu , J. Xiao , X. D. Su , Z. Du , Y. Feng , C. Qin , et al., Cell 2020, 182, 73; 32425270 10.1016/j.cell.2020.05.025 PMC7231725

[13] P. J. M. Brouwer , T. G. Caniels , K. van der Straten , J. L. Snitselaar , Y. Aldon , S. Bangaru , J. L. Torres , N. M. A. Okba , M. Claireaux , G. Kerster , A. E. H. Bentlage , M. M. van Haaren , D. Guerra , J. A. Burger , E. E. Schermer , K. D. Verheul , N. van der Velde , A. van der Kooi , J. van Schooten , M. J. van Breemen , T. P. L. Bijl , K. Sliepen , A. Aartse , R. Derking , I. Bontjer , N. A. Kootstra , W. J. Wiersinga , G. Vidarsson , B. L. Haagmans , A. B. Ward , et al., Science 2020, 369, 643; 32540902 10.1126/science.abc5902 PMC7299281

[14] K. M. Hastie , H. Li , D. Bedinger , S. L. Schendel , S. M. Dennison , K. Li , V. Rayaprolu , X. Yu , C. Mann , M. Zandonatti , R. Diaz Avalos , D. Zyla , T. Buck , S. Hui , K. Shaffer , C. Hariharan , J. Yin , E. Olmedillas , A. Enriquez , D. Parekh , M. Abraha , E. Feeney , G. Q. Horn , Y. Aldon , H. Ali , S. Aracic , R. R. Cobb , R. S. Federman , J. M. Fernandez , et al., Science 2021, 374, 472; 34554826 10.1126/science.abh2315 PMC9302186

[15] T. F. Rogers , F. Zhao , D. Huang , N. Beutler , A. Burns , W. T. He , O. Limbo , C. Smith , G. Song , J. Woehl , L. Yang , R. K. Abbott , S. Callaghan , E. Garcia , J. Hurtado , M. Parren , L. Peng , S. Ramirez , J. Ricketts , M. J. Ricciardi , S. A. Rawlings , N. C. Wu , M. Yuan , D. M. Smith , D. Nemazee , J. R. Teijaro , J. E. Voss , I. A. Wilson , R. Andrabi , B. Briney , et al., Science 2020, 369, 956. 32540903 10.1126/science.abc7520 PMC7299280

[16] R. Wang , Q. Zhang , J. Ge , W. Ren , R. Zhang , J. Lan , B. Ju , B. Su , F. Yu , P. Chen , H. Liao , Y. Feng , X. Li , X. Shi , Z. Zhang , F. Zhang , Q. Ding , T. Zhang , X. Wang , L. Zhang , Immunity 2021, 54, 1611; 34166623 10.1016/j.immuni.2021.06.003 PMC8185182

[17] Q. Wang , T. Ma , Y. Wu , Z. Chen , H. Zeng , Z. Tong , F. Gao , J. Qi , Z. Zhao , Y. Chai , H. Yang , G. Wong , Y. Bi , L. Wu , R. Shi , M. Yang , J. Song , H. Jiang , Z. An , J. Wang , T. D. Yilma , Y. Shi , W. J. Liu , M. Liang , C. Qin , G. F. Gao , J. Yan , Nat. Microbiol. 2019, 4, 1231. 30936489 10.1038/s41564-019-0411-z

[18] P. Rawat , D. Sharma , R. Prabakaran , F. Ridha , M. Mohkhedkar , V. Janakiraman , M. M. Gromiha , Bioinformatics 2022, 38, 4051. 35771624 10.1093/bioinformatics/btac439

[19] D. Wrapp , N. Wang , K. S. Corbett , J. A. Goldsmith , C. L. Hsieh , O. Abiona , B. S. Graham , J. S. McLellan , Science 2020, 367, 1260; 32075877 10.1126/science.abb2507 PMC7164637

[20] T. Zhou , L. Wang , J. Misasi , A. Pegu , Y. Zhang , D. R. Harris , A. S. Olia , C. A. Talana , E. S. Yang , M. Chen , M. Choe , W. Shi , I. T. Teng , A. Creanga , C. Jenkins , K. Leung , T. Liu , E. D. Stancofski , T. Stephens , B. Zhang , Y. Tsybovsky , B. S. Graham , J. R. Mascola , N. J. Sullivan , P. D. Kwong , Science 2022, 376, abn8897; 10.1126/science.abn8897 PMC9580340 35324257

[21] P. Liu , C. Yue , B. Meng , T. Xiao , S. Yang , S. Liu , F. Jian , Q. Zhu , Y. Yu , Y. Ren , P. Wang , Y. Li , J. Wang , X. Mao , F. Shao , Y. Wang , R. K. Gupta , Y. Cao , X. Wang , Natl. Sci. Rev. 2024, 11, nwae206. 39071099 10.1093/nsr/nwae206 PMC11282955

[22] H. M. Callaway , K. M. Hastie , S. L. Schendel , H. Li , X. Yu , J. Shek , T. Buck , S. Hui , D. Bedinger , C. Troup , S. M. Dennison , K. Li , M. D. Alpert , C. C. Bailey , S. Benzeno , J. L. Bonnevier , J. Q. Chen , C. Chen , H. Cho , P. D. Crompton , V. Dussupt , K. C. Entzminger , Y. Ezzyat , J. K. Fleming , N. Geukens , A. E. Gilbert , Y. Guan , X. Han , C. J. Harvey , J. M. Hatler , et al., Cell Rep. 2023, 42, 112014. 36681898 10.1016/j.celrep.2023.112014 PMC9834171

[23] V. Yin , S. H. Lai , T. G. Caniels , P. J. M. Brouwer , M. Brinkkemper , Y. Aldon , H. Liu , M. Yuan , I. A. Wilson , R. W. Sanders , M. J. van Gils , A. J. R. Heck , ACS Cent. Sci. 2021, 7, 1863; 34845440 10.1021/acscentsci.1c00804 PMC8577368

[24] D. Zhou , H. M. E. Duyvesteyn , C. P. Chen , C. G. Huang , T. H. Chen , S. R. Shih , Y. C. Lin , C. Y. Cheng , S. H. Cheng , Y. C. Huang , T. Y. Lin , C. Ma , J. Huo , L. Carrique , T. Malinauskas , R. R. Ruza , P. N. M. Shah , T. K. Tan , P. Rijal , R. F. Donat , K. Godwin , K. R. Buttigieg , J. A. Tree , J. Radecke , N. G. Paterson , P. Supasa , J. Mongkolsapaya , G. R. Screaton , M. W. Carroll , J. Gilbert‐Jaramillo , et al., Nat. Struct. Mol. Biol. 2020, 27, 950; 32737466 10.1038/s41594-020-0480-y

[25] W. Dejnirattisai , D. Zhou , H. M. Ginn , H. M. E. Duyvesteyn , P. Supasa , J. B. Case , Y. Zhao , T. S. Walter , A. J. Mentzer , C. Liu , B. Wang , G. C. Paesen , J. Slon‐Campos , C. López‐Camacho , N. M. Kafai , A. L. Bailey , R. E. Chen , B. Ying , C. Thompson , J. Bolton , A. Fyfe , S. Gupta , T. K. Tan , J. Gilbert‐Jaramillo , W. James , M. Knight , M. W. Carroll , D. Skelly , C. Dold , Y. Peng , et al., Cell 2021, 184, 2183. 33756110 10.1016/j.cell.2021.02.032 PMC7891125

[26] C. O. Barnes , A. P. West , K. E. Huey‐Tubman , M. A. G. Hoffmann , N. G. Sharaf , P. R. Hoffman , N. Koranda , H. B. Gristick , C. Gaebler , F. Muecksch , J. C. C. Lorenzi , S. Finkin , T. Hägglöf , A. Hurley , K. G. Millard , Y. Weisblum , F. Schmidt , T. Hatziioannou , P. D. Bieniasz , M. Caskey , D. F. Robbiani , M. C. Nussenzweig , P. J. Bjorkman , Cell 2020, 182, 828. 32645326 10.1016/j.cell.2020.06.025 PMC7311918

[27] X. Nan , Y. Li , R. Zhang , R. Wang , N. Lv , J. Li , Y. Chen , B. Zhou , Y. Wang , Z. Wang , J. Zhu , J. Chen , J. Li , W. Chen , Q. Zhang , X. Shi , C. Zhao , C. Chen , Z. Liu , Y. Zhao , D. Liu , X. Wang , L. T. Yan , T. Li , L. Zhang , Y. R. Yang , Nat. Commun. 2024, 15, 10578; 39632831 10.1038/s41467-024-54746-5 PMC11618796

[28] D. Pinto , Y.‐J. Park , M. Beltramello , A. C. Walls , M. A. Tortorici , S. Bianchi , S. Jaconi , K. Culap , F. Zatta , A. De Marco , A. Peter , B. Guarino , R. Spreafico , E. Cameroni , J. B. Case , R. E. Chen , C. Havenar‐Daughton , G. Snell , A. Telenti , H. W. Virgin , A. Lanzavecchia , M. S. Diamond , K. Fink , D. Veesler , D. Corti , Nature 2020, 583, 290; 32422645 10.1038/s41586-020-2349-y

[29] D. Asarnow , B. Wang , W. H. Lee , Y. Hu , C. W. Huang , B. Faust , P. M. L. Ng , E. Z. X. Ngoh , M. Bohn , D. Bulkley , A. Pizzorno , B. Ary , H. C. Tan , C. Y. Lee , R. A. Minhat , O. Terrier , M. K. Soh , F. J. Teo , Y. Y. C. Yeap , S. G. K. Seah , C. E. Z. Chan , E. Connelly , N. J. Young , S. Maurer‐Stroh , L. Renia , B. J. Hanson , M. Rosa‐Calatrava , A. Manglik , Y. Cheng , C. S. Craik , et al., Cell 2021, 184, 3192. 33974910 10.1016/j.cell.2021.04.033 PMC8064868

[30] D. S. Khoury , A. K. Wheatley , M. D. Ramuta , A. Reynaldi , D. Cromer , K. Subbarao , D. H. O'Connor , S. J. Kent , M. P. Davenport , Nat. Rev. Immunol. 2020, 20, 727. 33139888 10.1038/s41577-020-00471-1 PMC7605490

[31] H. Yang , Z. Rao , Nat. Rev. Microbiol. 2021, 19, 685. 34535791 10.1038/s41579-021-00630-8 PMC8447893

[32] B. Ju , Q. Zhang , Z. Wang , Z. Q. Aw , P. Chen , B. Zhou , R. Wang , X. Ge , Q. Lv , L. Cheng , R. Zhang , Y. H. Wong , H. Chen , H. Wang , S. Shan , X. Liao , X. Shi , L. Liu , J. J. H. Chu , X. Wang , Z. Zhang , L. Zhang , Nat. Immunol. 2023, 24, 690; 36914890 10.1038/s41590-023-01449-6 PMC10063446

[33] Q. Zhang , B. Ju , J. Ge , J. F. Chan , L. Cheng , R. Wang , W. Huang , M. Fang , P. Chen , B. Zhou , S. Song , S. Shan , B. Yan , S. Zhang , X. Ge , J. Yu , J. Zhao , H. Wang , L. Liu , Q. Lv , L. Fu , X. Shi , K. Y. Yuen , L. Liu , Y. Wang , Z. Chen , L. Zhang , X. Wang , Z. Zhang , Nat. Commun. 2021, 12, 4210; 34244522 10.1038/s41467-021-24514-w PMC8270942

[34] M. Yuan , H. Liu , N. C. Wu , C. D. Lee , X. Zhu , F. Zhao , D. Huang , W. Yu , Y. Hua , H. Tien , T. F. Rogers , E. Landais , D. Sok , J. G. Jardine , D. R. Burton , I. A. Wilson , Science 2020, 369, 1119; 32661058 10.1126/science.abd2321 PMC7402627

[35] J. Ge , R. Wang , B. Ju , Q. Zhang , J. Sun , P. Chen , S. Zhang , Y. Tian , S. Shan , L. Cheng , B. Zhou , S. Song , J. Zhao , H. Wang , X. Shi , Q. Ding , L. Liu , J. Zhao , Z. Zhang , X. Wang , L. Zhang , Nat. Commun. 2021, 12, 250; 33431856 10.1038/s41467-020-20501-9 PMC7801515

[36] Y. Wu , F. Wang , C. Shen , W. Peng , D. Li , C. Zhao , Z. Li , S. Li , Y. Bi , Y. Yang , Y. Gong , H. Xiao , Z. Fan , S. Tan , G. Wu , W. Tan , X. Lu , C. Fan , Q. Wang , Y. Liu , C. Zhang , J. Qi , G. F. Gao , F. Gao , L. Liu , Science 2020, 368, 1274; 32404477 10.1126/science.abc2241 PMC7223722

[37] H. Liu , N. C. Wu , M. Yuan , S. Bangaru , J. L. Torres , T. G. Caniels , J. van Schooten , X. Zhu , C. D. Lee , P. J. M. Brouwer , M. J. van Gils , R. W. Sanders , A. B. Ward , I. A. Wilson , Immunity 2020, 53, 1272; 33242394 10.1016/j.immuni.2020.10.023 PMC7687367

[38] N. K. Hurlburt , E. Seydoux , Y. H. Wan , V. V. Edara , A. B. Stuart , J. Feng , M. S. Suthar , A. T. McGuire , L. Stamatatos , M. Pancera , Nat. Commun. 2020, 11, 5413. 33110068 10.1038/s41467-020-19231-9 PMC7591918

[39] C. O. Barnes , C. A. Jette , M. E. Abernathy , K. A. Dam , S. R. Esswein , H. B. Gristick , A. G. Malyutin , N. G. Sharaf , K. E. Huey‐Tubman , Y. E. Lee , D. F. Robbiani , M. C. Nussenzweig , A. P. West Jr. , P. J. Bjorkman , Nature 2020, 588, 682; 33045718 10.1038/s41586-020-2852-1 PMC8092461

[40] L. Liu , P. Wang , M. S. Nair , J. Yu , M. Rapp , Q. Wang , Y. Luo , J. F. Chan , V. Sahi , A. Figueroa , X. V. Guo , G. Cerutti , J. Bimela , J. Gorman , T. Zhou , Z. Chen , K. Y. Yuen , P. D. Kwong , J. G. Sodroski , M. T. Yin , Z. Sheng , Y. Huang , L. Shapiro , D. D. Ho , Nature 2020, 584, 450; 32698192 10.1038/s41586-020-2571-7

[41] F. Muecksch , Y. Weisblum , C. O. Barnes , F. Schmidt , D. Schaefer‐Babajew , Z. Wang , C. L. J. C. , A. I. Flyak , A. T. DeLaitsch , K. E. Huey‐Tubman , S. Hou , C. A. Schiffer , C. Gaebler , J. D. Silva , D. Poston , S. Finkin , A. Cho , M. Cipolla , T. Y. Oliveira , K. G. Millard , V. Ramos , A. Gazumyan , M. Rutkowska , M. Caskey , M. C. Nussenzweig , P. J. Bjorkman , T. Hatziioannou , P. D. Bieniasz , Immunity 2021, 54, 1853; 34331873 10.1016/j.immuni.2021.07.008 PMC8323339

[42] M. Luo , R. Zhou , B. Tang , H. Liu , B. Chen , N. Liu , Y. Mo , P. Zhang , Y. L. Lee , J. D. Ip , A. Wing‐Ho Chu , W. M. Chan , H. O. Man , Y. Chen , K. K. To , K. Y. Yuen , S. Dang , Z. Chen , EBioMedicine 2024, 108, 105354; 39341153 10.1016/j.ebiom.2024.105354 PMC11470419

[43] Y. J. Park , A. De Marco , T. N. Starr , Z. Liu , D. Pinto , A. C. Walls , F. Zatta , S. K. Zepeda , J. E. Bowen , K. R. Sprouse , A. Joshi , M. Giurdanella , B. Guarino , J. Noack , R. Abdelnabi , S. C. Foo , L. E. Rosen , F. A. Lempp , F. Benigni , G. Snell , J. Neyts , S. P. J. Whelan , H. W. Virgin , J. D. Bloom , D. Corti , M. S. Pizzuto , D. Veesler , Science 2022, 375, 449; 34990214 10.1126/science.abm8143 PMC9400459

[44] M. A. Tortorici , M. Beltramello , F. A. Lempp , D. Pinto , H. V. Dang , L. E. Rosen , M. McCallum , J. Bowen , A. Minola , S. Jaconi , F. Zatta , A. De Marco , B. Guarino , S. Bianchi , E. J. Lauron , H. Tucker , J. Zhou , A. Peter , C. Havenar‐Daughton , J. A. Wojcechowskyj , J. B. Case , R. E. Chen , H. Kaiser , M. Montiel‐Ruiz , M. Meury , N. Czudnochowski , R. Spreafico , J. Dillen , C. Ng , N. Sprugasci , et al., Science 2020, 370, 950; 32972994 10.1126/science.abe3354 PMC7857395

[45] Z. Lv , Y. Q. Deng , Q. Ye , L. Cao , C. Y. Sun , C. Fan , W. Huang , S. Sun , Y. Sun , L. Zhu , Q. Chen , N. Wang , J. Nie , Z. Cui , D. Zhu , N. Shaw , X. F. Li , Q. Li , L. Xie , Y. Wang , Z. Rao , C. F. Qin , X. Wang , Science 2020, 369, 1505; 32703908 10.1126/science.abc5881 PMC7402622

[46] X. Chi , R. Yan , J. Zhang , G. Zhang , Y. Zhang , M. Hao , Z. Zhang , P. Fan , Y. Dong , Y. Yang , Z. Chen , Y. Guo , J. Zhang , Y. Li , X. Song , Y. Chen , L. Xia , L. Fu , L. Hou , J. Xu , C. Yu , J. Li , Q. Zhou , W. Chen , Science 2020, 369, 650; 32571838 10.1126/science.abc6952 PMC7319273

[47] Y. Wang , C. Liu , C. Zhang , Y. Wang , Q. Hong , S. Xu , Z. Li , Y. Yang , Z. Huang , Y. Cong , Nat. Commun. 2022, 13, 871; 35169135 10.1038/s41467-022-28528-w PMC8847413

[48] S. Moriyama , Y. Anraku , S. Taminishi , Y. Adachi , D. Kuroda , S. Kita , Y. Higuchi , Y. Kirita , R. Kotaki , K. Tonouchi , K. Yumoto , T. Suzuki , T. Someya , H. Fukuhara , Y. Kuroda , T. Yamamoto , T. Onodera , S. Fukushi , K. Maeda , F. Nakamura‐Uchiyama , T. Hashiguchi , A. Hoshino , K. Maenaka , Y. Takahashi , Nat. Commun. 2023, 14, 4198; 37452031 10.1038/s41467-023-39890-8 PMC10349087

[49] F. Jian , A. Z. Wec , L. Feng , Y. Yu , L. Wang , P. Wang , L. Yu , J. Wang , J. Hou , D. M. Berrueta , D. Lee , T. Speidel , L. Ma , T. Kim , A. Yisimayi , W. Song , J. Wang , L. Liu , S. Yang , X. Niu , T. Xiao , R. An , Y. Wang , F. Shao , Y. Wang , S. Pecetta , X. Wang , L. M. Walker , Y. Cao , Nat. Microbiol. 2025, 10, 2003; 40494884 10.1038/s41564-025-02030-7 PMC12313522

[50] N. Li , Z. Li , Y. Fu , S. Cao , Virol. Sin. 2020, 35, 1. 31916022 10.1007/s12250-019-00190-5 PMC7035235

[51] M. Yuan , H. Liu , N. C. Wu , I. A. Wilson , Biochem. Biophys. Res. Commun. 2021, 538, 192. 33069360 10.1016/j.bbrc.2020.10.012 PMC7547570

[52] H. Yao , Y. Sun , Y.‐Q. Deng , N. Wang , Y. Tan , N.‐N. Zhang , X.‐F. Li , C. Kong , Y.‐P. Xu , Q. Chen , T.‐S. Cao , H. Zhao , X. Yan , L. Cao , Z. Lv , D. Zhu , R. Feng , N. Wu , W. Zhang , Y. Hu , K. Chen , R.‐R. Zhang , Q. Lv , S. Sun , Y. Zhou , R. Yan , G. Yang , X. Sun , C. Liu , X. Lu , et al., Cell Res. 2020, 31, 25.33262452

[53] H. Yao , Y. Song , Q. Huang , M. Zhu , J. Liang , Z. Zhang , X. Zhang , D. Dong , D. Shi , Z. Wu , X. Lu , H. Wu , Y. Chen , S. Li , bioRxiv 2025.

[54] C. A. Diebolder , F. J. Beurskens , R. N. de Jong , R. I. Koning , K. Strumane , M. A. Lindorfer , M. Voorhorst , D. Ugurlar , S. Rosati , A. J. Heck , J. G. van de Winkel , I. A. Wilson , A. J. Koster , R. P. Taylor , E. O. Saphire , D. R. Burton , J. Schuurman , P. Gros , P. W. Parren , Science 2014, 343, 1260. 24626930 10.1126/science.1248943 PMC4250092

[55] D. Ghimire , Y. Han , M. Lu , Viruses 2022, 14, 1255. 35746726 10.3390/v14061255 PMC9229035

[56] Y. Liao , H. Ma , Z. Wang , S. Wang , Y. He , Y. Chang , H. Zong , H. Tang , L. Wang , Y. Ke , H. Cai , P. Li , J. Tang , H. Chen , A. Drelich , B. H. Peng , J. Hsu , V. Tat , C. K. Tseng , J. Song , Y. Yuan , M. Wu , J. Liu , Y. Yue , X. Zhang , Z. Wang , L. Yang , J. Li , X. Ni , H. Li , et al., Proc. Natl. Acad. Sci. USA 2025, 122, 2406659122.

[57] S. Musiu , Y. P. Castillo , A. Muigg , G. Purstinger , P. Leyssen , M. Froeyen , J. Neyts , J. Paeshuyse , Molecules 2020, 25, 1283; 32178258 10.3390/molecules25061283 PMC7144022

[58] T. Ni , M. Yuan , H. H. Ji , G. Tang , Y. Chen , Z. Ma , X. D. Li , ACS Omega 2020, 5, 21815. 32905433 10.1021/acsomega.0c02886 PMC7469408

[59] W. B. Wang , Y. B. Ma , Z. H. Lei , X. F. Zhang , J. Li , S. S. Li , Z. Y. Dong , Y. Liang , Q. M. Li , J. G. Su , J. Mol. Graph. Model. 2023, 124, 108540. 37352723 10.1016/j.jmgm.2023.108540 PMC10254043

[60] G. A. Voth , Coarse‐graining of condensed phase and biomolecular systems, CRC Press, Boca Raton, FL 2008.

[61] Y. Cai , J. Zhang , T. Xiao , H. Peng , S. M. Sterling , R. M. Walsh Jr. , S. Rawson , S. Rits‐Volloch , B. Chen , Science 2020, 369, 1586. 32694201 10.1126/science.abd4251 PMC7464562

[62] E. Słyk , T. Skóra , S. Kondrat , J. Phys. Chem. B 2023, 127, 7442. 37591305 10.1021/acs.jpcb.3c02383 PMC10476189

[63] J. W. Jay , B. Bray , Y. Qi , E. Igbinigie , H. Wu , J. Li , G. Ren , Antibodies 2018, 7, 18. 31544870 10.3390/antib7020018 PMC6698877

[64] Q. He , Y. An , X. Zhou , H. Xie , L. Tao , D. Li , A. Zheng , L. Li , Z. Xu , S. Yu , R. Wang , H. Hu , K. Liu , Q. Wang , L. Dai , K. Xu , G. F. Gao , Med 2024, 5, 401. 38574739 10.1016/j.medj.2024.03.006

[65] J. Homol , Chem. Rev. 2008, 108, 462; 18229953 10.1021/cr068107d

[66] L. Liu , C. Han , M. Jiang , T. Zhang , Q. Kang , X. Wang , P. Wang , F. Zhou , Anal. Chim. Acta. 2021, 1170, 338625. 34090589 10.1016/j.aca.2021.338625

[67] H. H. Nguyen , J. Park , S. Kang , M. Kim , Sensors 2015, 15, 10481. 25951336 10.3390/s150510481 PMC4481982

[68] P. D. Adams , P. V. Afonine , G. Bunkóczi , V. B. Chen , I. W. Davis , N. Echols , J. J. Headd , L.‐W. Hung , G. J. Kapral , R. W. Grosse‐Kunstleve , A. J. McCoy , N. W. Moriarty , R. Oeffner , R. J. Read , D. C. Richardson , J. S. Richardson , T. C. Terwilliger , P. H. Zwart , Acta Crystallogr. Sect. D: Biol. Crystallogr. 2010, 66, 213. 20124702 10.1107/S0907444909052925 PMC2815670

[69] P. Emsley , B. Lohkamp , W. G. Scott , K. Cowtan , Acta Crystallogr. Sect. D: Biol. Crystallogr. 2010, 66, 486. 20383002 10.1107/S0907444910007493 PMC2852313

[70] E. F. Pettersen , T. D. Goddard , C. C. Huang , G. S. Couch , D. M. Greenblatt , E. C. Meng , T. E. Ferrin , J. Comput. Chem. 2004, 25, 1605. 15264254 10.1002/jcc.20084

[71] R. Wang , Y. Han , R. Zhang , J. Zhu , X. Nan , Y. Liu , Z. Yang , B. Zhou , J. Yu , Z. Lin , J. Li , P. Chen , Y. Wang , Y. Li , D. Liu , X. Shi , X. Wang , Q. Zhang , Y. R. Yang , T. Li , L. Zhang , Immunity 2023, 56, 2635. 37924813 10.1016/j.immuni.2023.10.007

[72] K. Zhang , J. Struct. Biol. 2016, 193, 1. 26592709 10.1016/j.jsb.2015.11.003 PMC4711343

[73] A. Punjani , J. L. Rubinstein , D. J. Fleet , M. A. Brubaker , Nat. Methods 2017, 14, 290. 28165473 10.1038/nmeth.4169

[74] Z. Liu , R. Guo , G. Xu , Z. Huang , L.‐T. Yan , Nano Lett. 2014, 14, 6910; 25375409 10.1021/nl5029396

[75] X. Li , X. Dai , Y. Pan , Y. Sun , B. Yang , K. Chen , Y. Wang , J.‐F. Xu , Y. Dong , Y. R. Yang , L.‐T. Yan , D. Liu , J. Am. Chem. Soc. 2022, 144, 21267. 36374167 10.1021/jacs.2c08930

[76] Y.‐L. Zhu , H. Liu , Z.‐W. Li , H.‐J. Qian , G. Milano , Z.‐Y. Lu , J. Comput. Chem. 2013, 34, 2197. 24137668 10.1002/jcc.23365

[77] P. Virtanen , R. Gommers , T. E. Oliphant , M. Haberland , T. Reddy , D. Cournapeau , E. Burovski , P. Peterson , W. Weckesser , J. Bright , S. J. van der Walt , M. Brett , J. Wilson , K. J. Millman , N. Mayorov , A. R. J. Nelson , E. Jones , R. Kern , E. Larson , C. J. Carey , I. Polat , Y. Feng , E. W. Moore , J. VanderPlas , D. Laxalde , J. Perktold , R. Cimrman , I. Henriksen , E. A. Quintero , C. R. Harris , et al., Nat. Methods 2020, 17, 261. 32015543 10.1038/s41592-019-0686-2 PMC7056644

[78] A. Paszke , S. Gross , F. Massa , A. Lerer , J. Bradbury , G. Chanan , T. Killeen , Z. Lin , N. Gimelshein , L. Antiga , A. Desmaison , A. Köpf , E. Yang , Z. DeVito , M. Raison , A. Tejani , S. Chilamkurthy , B. Steiner , L. Fang , J. Bai , S. Chintala , Advances in Neural Information Processing Systems 2019, 32, 8024.

[79] F. Pedregosa , G. Varoquaux , A. Gramfort , V. Michel , B. Thirion , O. Grisel , M. Blondel , P. Prettenhofer , R. Weiss , V. Dubourg , J. Vanderplas , A. Passos , D. Cournapeau , M. Brucher , M. Perrot , J. Mach. Learn. Res. 2011, 12, 2825.

