# Supplementary material for: IgG‐Bridging–Seeded Synergistic Aggregation of SARS‐CoV‐2 Spikes Underlies Potent Neutralization by a Low‐Affinity Antibody
Source: Adv Sci (Weinh). 2025 Dec 7;13(11):e17192. doi: 10.1002/advs.202517192 (PMC12931240; doi:10.1002/advs.202517192)
Supplement: Supplementary file 1 — Supporting Information [file ADVS-13-e17192-s002.docx]

Supporting Information

**IgG-Bridging–Seeded Synergistic Aggregation of SARS-CoV-2 Spikes Underlies Potent Neutralization by A Low-Affinity Antibody**

*Niannian Lv,^#^ Peng Chen,^#^ Xiaobin Dai,^#^ Hu Xu, Ziheng Li, Zelin Shan, Jinqian Li, Fenglin Guo, Yuanfang Chen, Jiayi Li, Yiqian Huang, Guizhi Dong, Yifan Jiang, Liang Chen, Xuanyu Nan, Hanjun Zhao, Kang Zhang, Shilong Fan, Yuanchen Dong, Dongsheng Liu, Xinquan Wang, Deli Huang, Xiaojing Pan, Chunying Chen, Zhihua Liu,* Li-Tang Yan,* Qi Zhang,* Linqi Zhang,* Yuliang Zhao,* Yuhe Renee Yang**


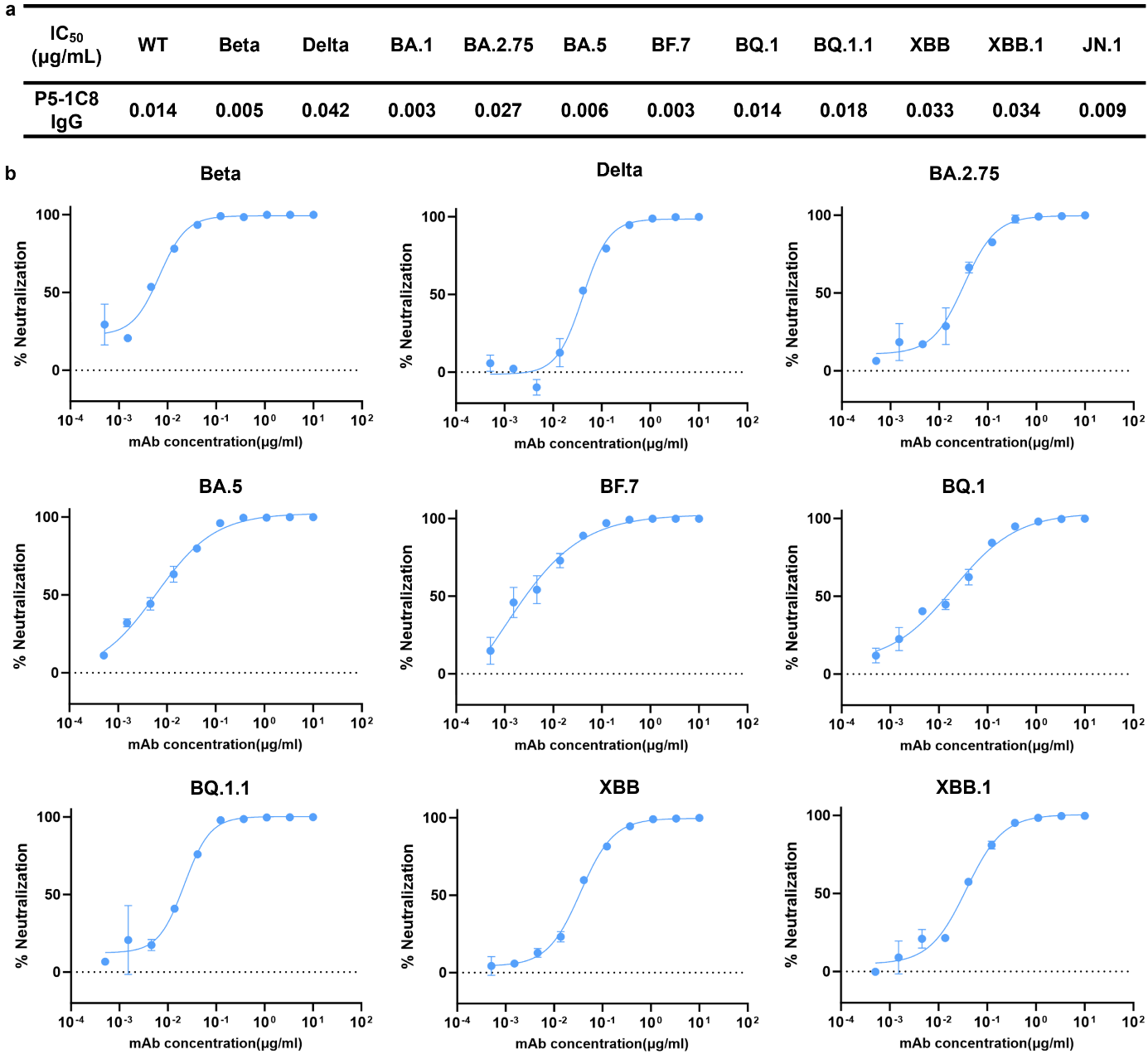
**Figure S1.** Neutralizing capability of P5-1C8 IgG against SARS-CoV-2 variants. a) Neutralizing potency (IC_50_) and neutralization curves (b) of P5-1C8 IgG against a panel of nine coronaviruses, with mean values and standard errors (s.e.m.) indicated. The detection limit, defined by the highest antibody concentration used, was 10 μg/mL. Data was obtained from two independent experiments, each performed with two technical replicates.


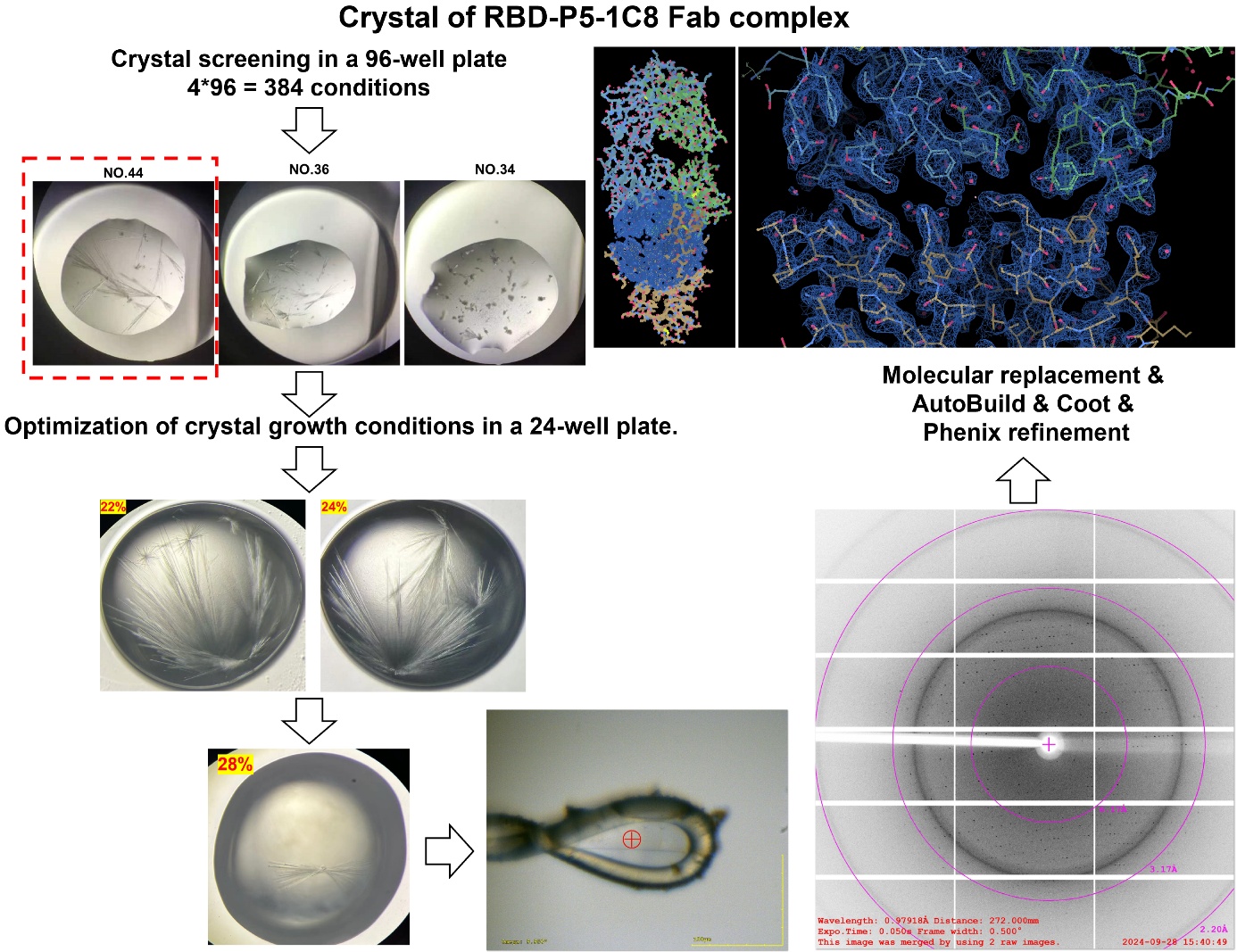
**Figure S2.** Workflow for crystallization optimization, X-ray data collection, and processing of the SARS-CoV-2 wild-type RBD in complex with P5-1C8 Fab.


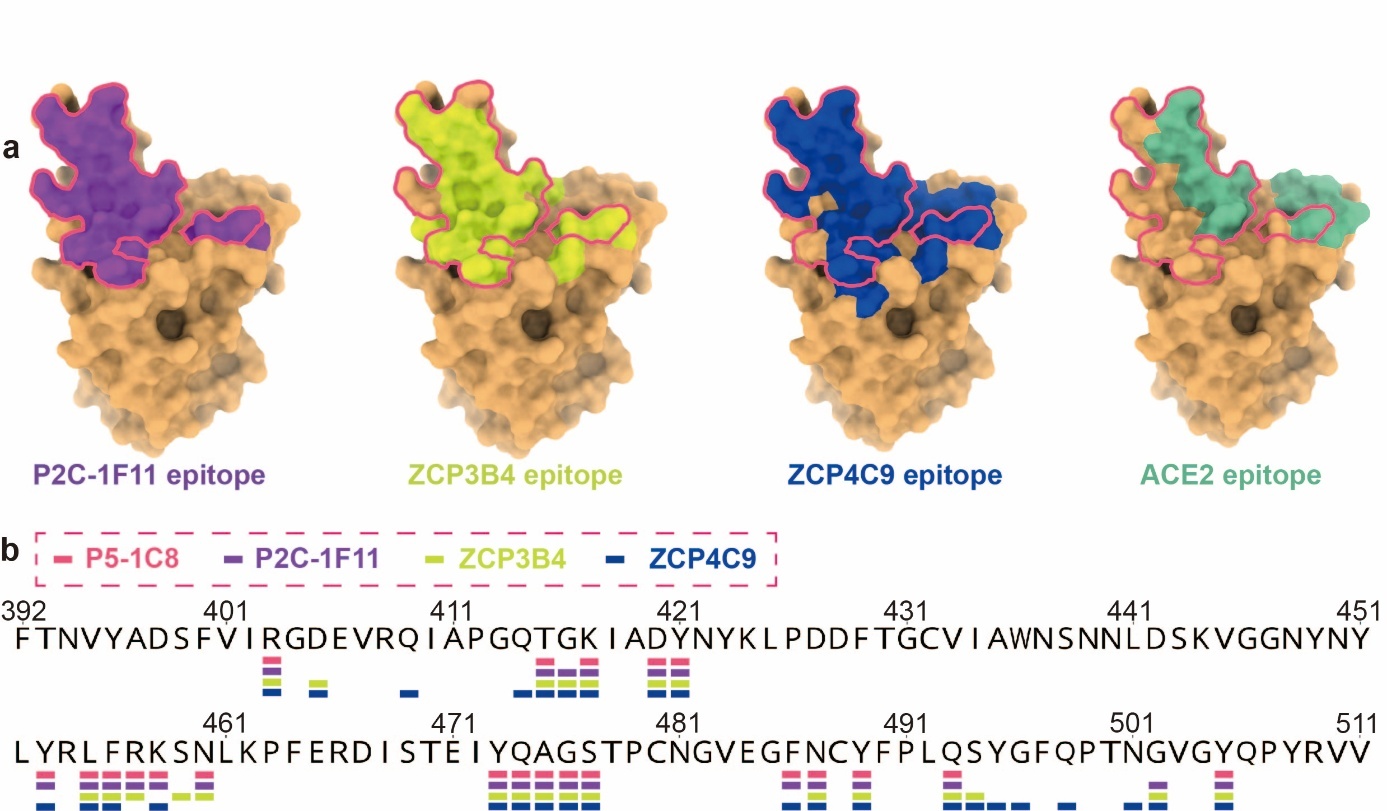
**Figure S3.** Epitope mapping of four class 1 antibodies and ACE2. a) The footprints of three Fabs and ACE2 on SARS-CoV-2 RBD. Dark violet, moderate yellow, blue, and green represent the footprint of P2C-1F11 Fab, ZCP3B4 Fab, ZCP4C9 Fab, and ACE2 respectively. b) RBD sequence from residues 392 to 511, with binding interface residues of the four antibodies highlighted by pink, dark violet, moderate yellow, and blue boxes, respectively.


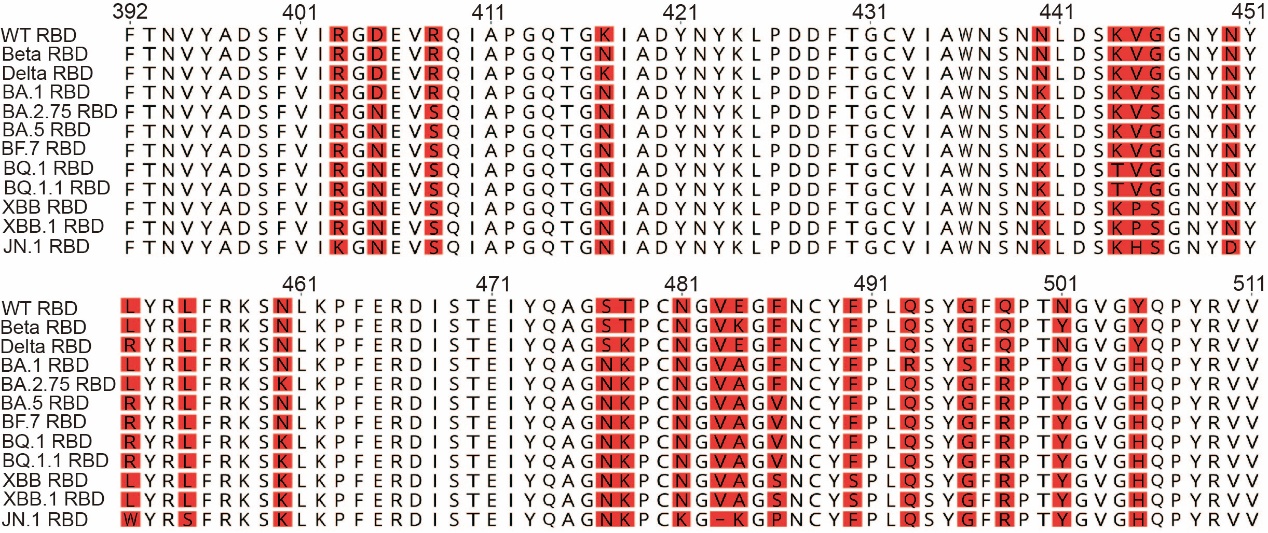
**Figure S4.** Sequence alignment of RBDs from SARS-CoV-2 variants included in the neutralization assays. Amino acids residues differing from the WT RBD are highlighted in red. The RBD sequences correspond exactly to the variant panel used in neutralization assays, including WT, Beta, Delta, BA.1, BA.2.75, BA.5, BF.7, BQ.1, BQ.1.1, XBB, XBB.1, and JN.1.


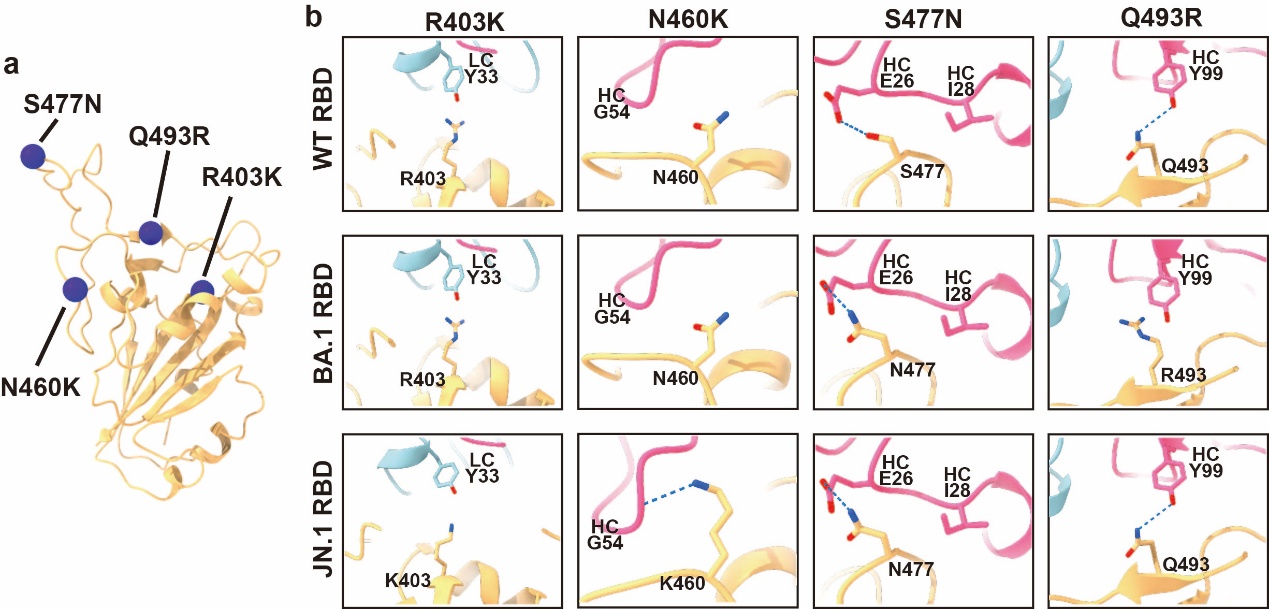
**Figure S5.** The interactions between P5-1C8 Fab and non-conserved residues in the RBD show only minor changes. a) Structure of the SARS-CoV-2 WT RBD, with key P5-1C8 Fab-binding residues highlighted as violet spheres. b) Shown are the structural comparisons of P5-1C8 Fab interactions with WT RBD (upper panel) and Omicron BA.1 RBD (middle panel), and JN.1 RBD (lower panel) at positions R403K, N460K, S477N, and Q493R. Contacting residues are depicted as sticks, and hydrogen bonds or salt bridges are indicated by dashed lines.


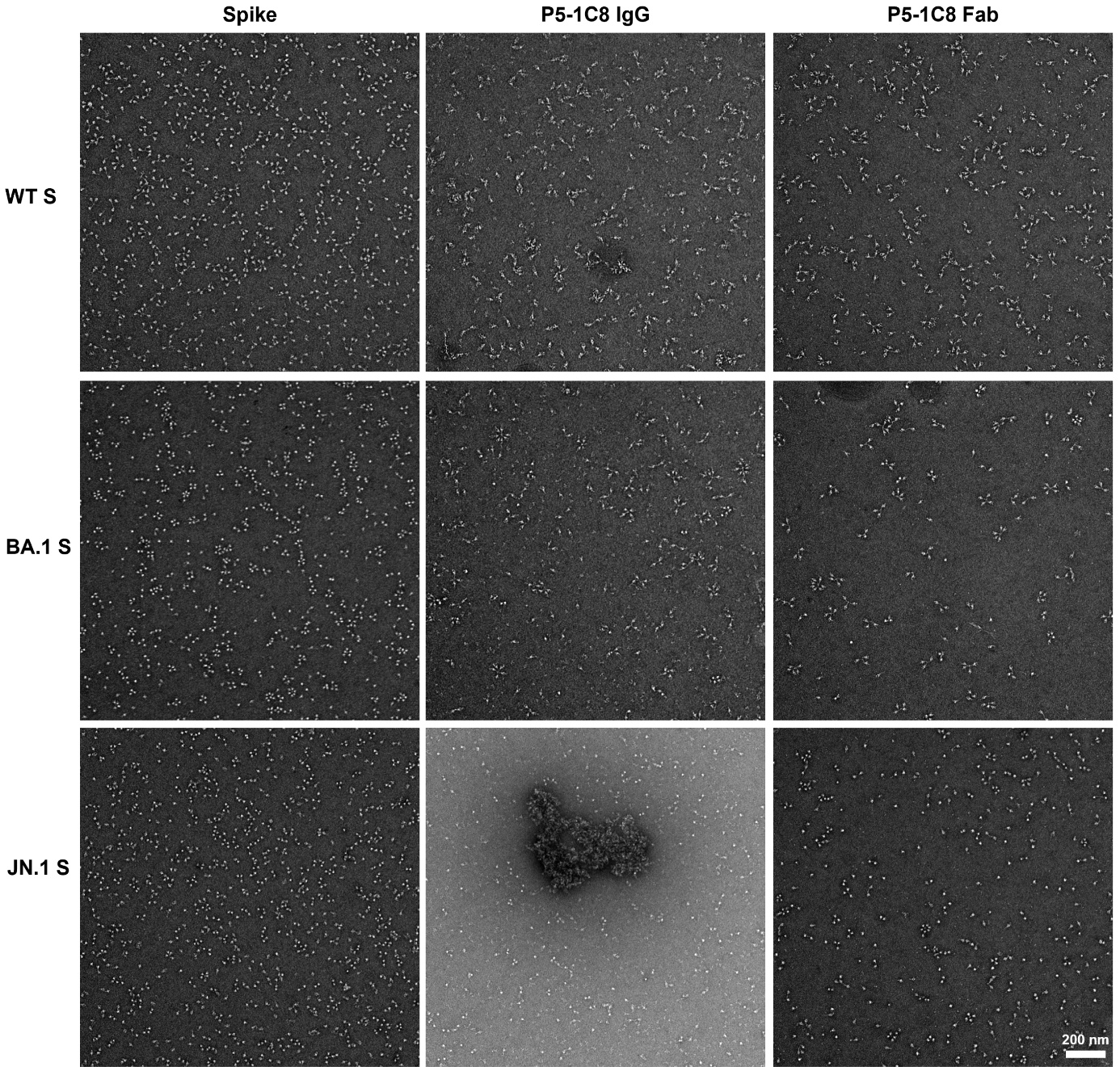
**Figure S6.** Negative-stain micrographs of SARS-CoV-2 spike trimers alone or in complex with P5-1C8 IgG or Fab for WT, BA.1, and JN.1 S. Prior to antibody addition, each soluble spikes were confirmed to be well-dispersed with no detectable aggregation. WT, BA.1, and JN.1 spike trimers were incubated with 3-fold molar excess of P5-1C8 IgG or Fab at RT for 1 h. Scale bar: 200 nm. The scale bar in the last image is applicable to all other images in the same panel.


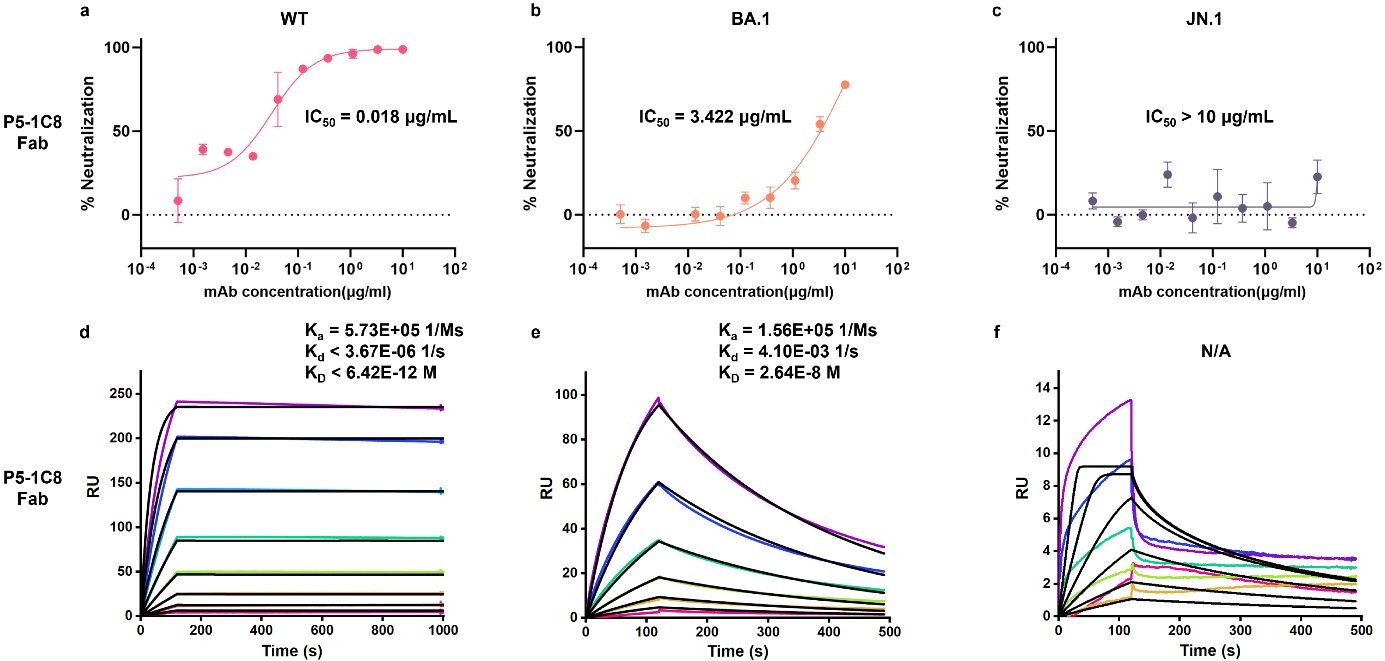
**Figure S7.** Neutralization potency and binding affinity of P5-1C8 Fab. a-c) Neutralization curves of P5-1C8 Fab against SARS-CoV-2 WT (a), BA.1 (b), and JN.1 (c) variants. Data was obtained from two independent experiments, each performed with two technical replicates. d-f) Binding kinetics between P5-1C8 Fab and the spike trimer of SARS-CoV-2 WT (d), BA.1 (e), and JN.1 (f), measured by surface plasmon resonance (SPR). Spike trimers were immobilized on a nitrilotriacetic acid (NTA) sensor chip, and serial dilutions of P5-1C8 Fab were flowed through the system. Colored lines represent experimentally measured sensorgrams. Black lines show the best-fit curves based on experimental data. The calculated association rate (K_a_), dissociation rate (K_d_), and equilibrium dissociation constant (K_D_) for each antibody-spike pair are indicated. All results were confirmed in three independent experiments.


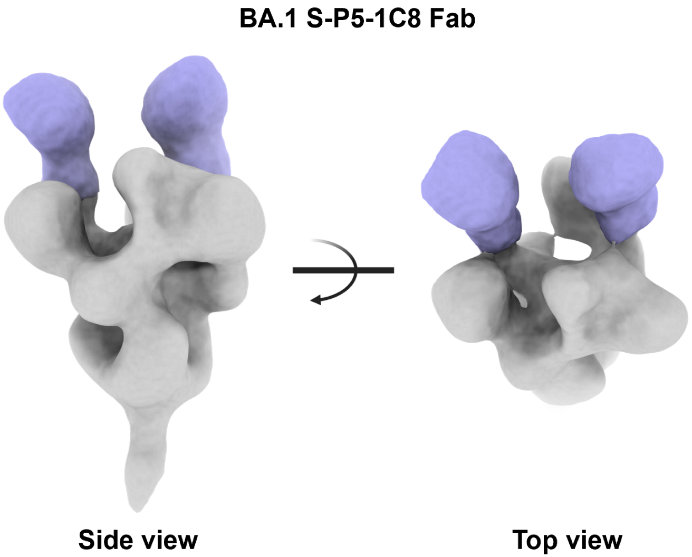
**Figure S8.** Representative 3D reconstructions of Omicron BA.1 spike trimers incubated with P5-1C8 Fab at a 3:1 Fab-to-protomer molar ratio, showing two Fab molecules bound per trimer.


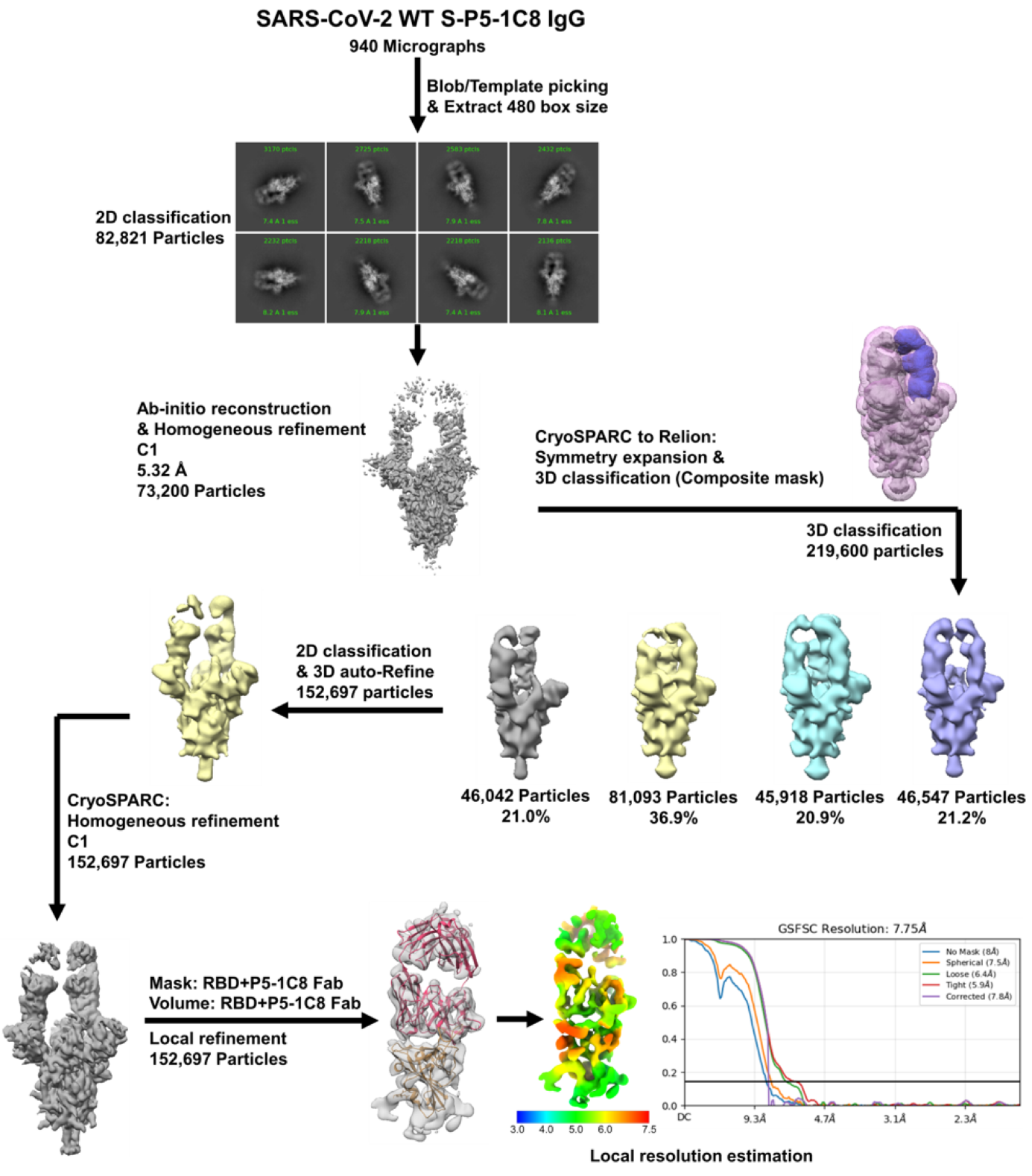
**Figure S9.** Cryo-EM data processing workflow for the SARS-CoV-2 WT spike protein in complex with P5-1C8 IgG.


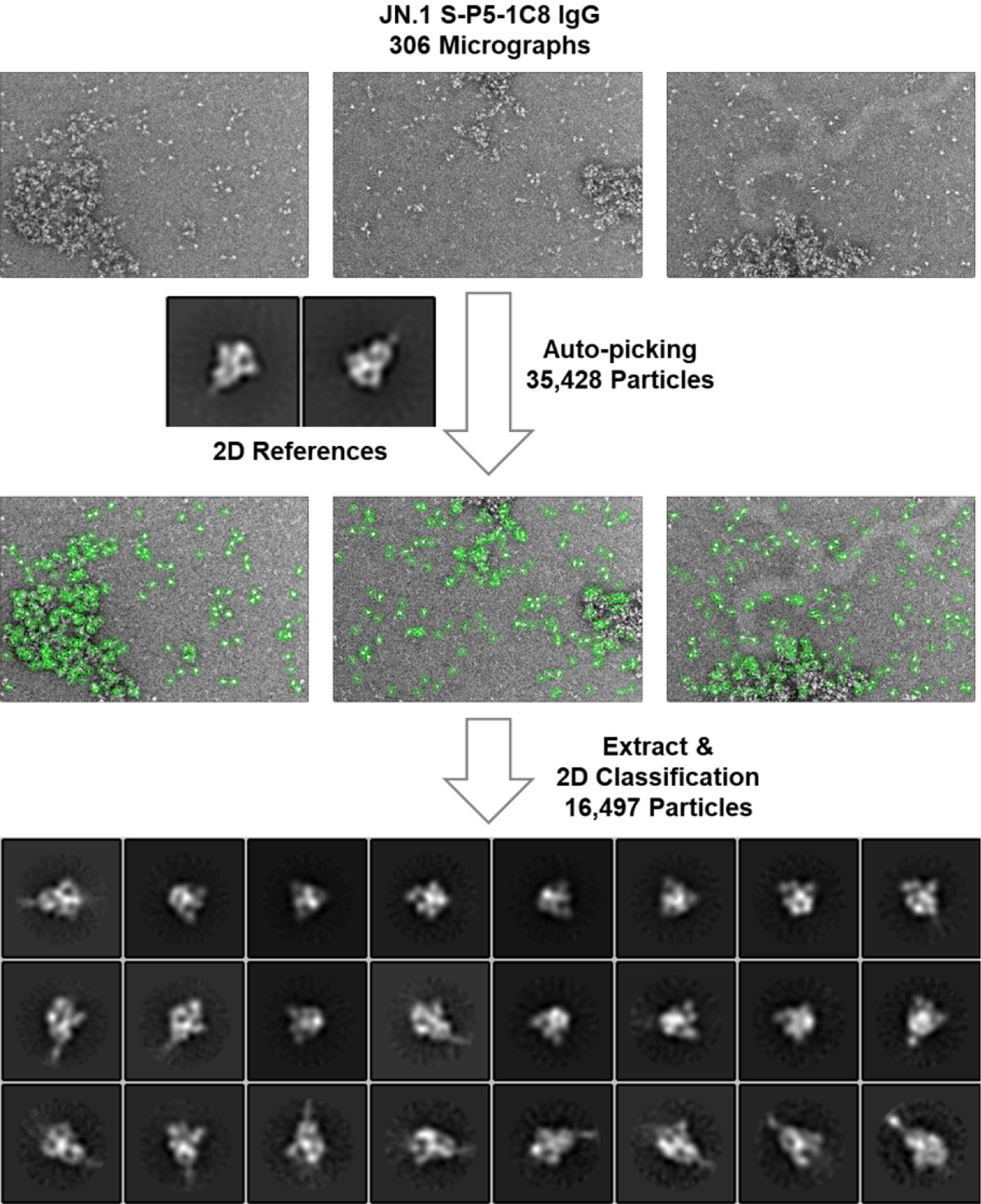
**Figure S10.** Auto-picking and data processing workflow for the JN.1 S-P5-1C8 IgG complex using Relion. Spike only were used as references during auto-picking to select non-aggregated particles. However, 2D class averages showed no detectable IgG density on the spike.


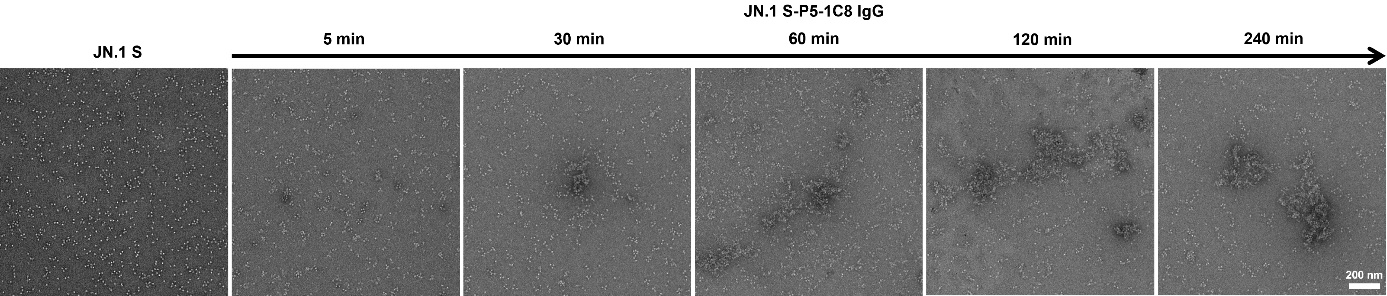
**Figure S11.** Negative-stain EM images of P5-1C8 IgG in complex with JN.1 spike trimers after incubation for 5 to 240 mins. Scale bar: 200 nm. The scale bar in the last image is applicable to all other images in the same panel.


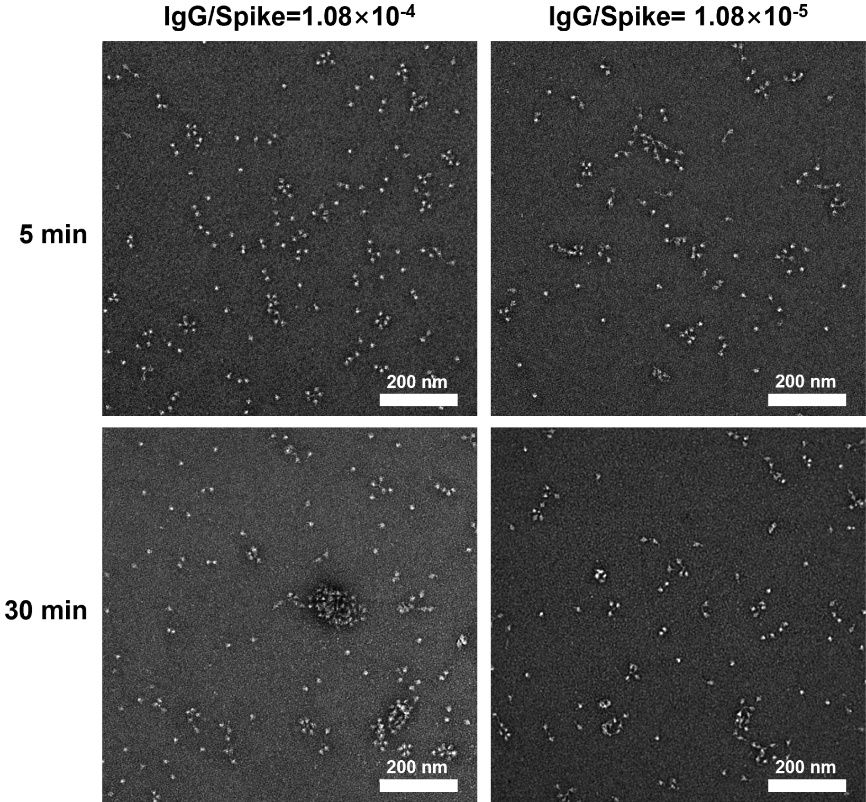
**Figure S12.** Negative-stain EM images of P5-1C8 IgG in complex with JN.1 spike trimers at IgG-to-spike molar ratio ranging from 1.08 × 10^-4^ to 1.08×10^-5^, following incubation for 5 to 30 min. An IgG/Spike ratio of 1.08 × 10^-4^ corresponds to an IgG concentration of 0.06 nM, approximating the IC_50_. Scale bar: 200 nm.


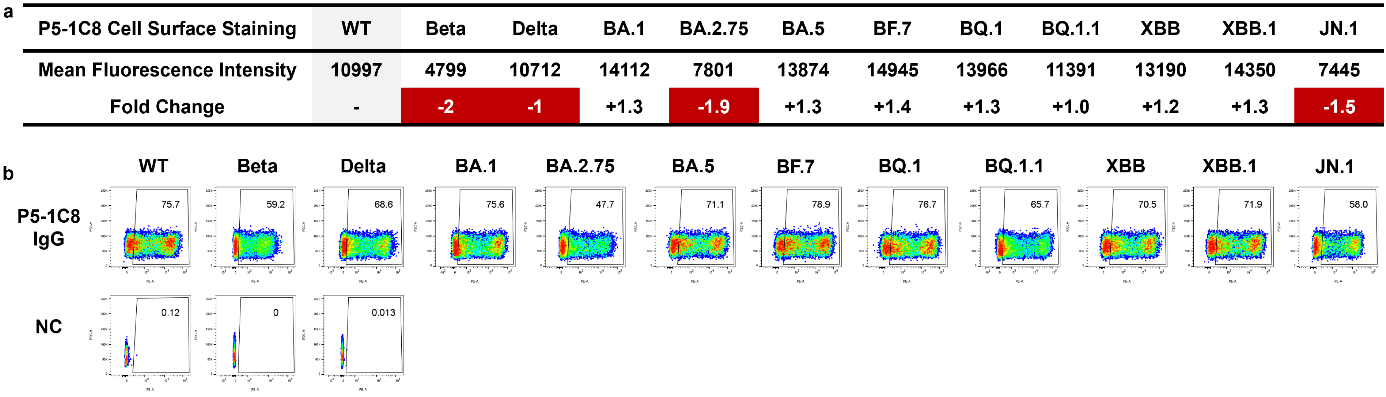
**Figure S13.** Cross-binding ability of P5-1C8 IgG to SARS-CoV-2 variants assessed by cell surface staining. a) For cell surface staining, spike proteins from diverse SARS-CoV-2 variants were expressed on the surface of HEK293T cells, stained with P5-1C8 IgG, and analyzed by flow cytometry. Total fluorescence intensity of positively stained cells was quantified and normalized to represent relative binding ability. Fold change was calculated relative to WT. b) Gating strategies and representative flow cytometry results for P5-1C8 IgG cell surface staining. Numbers in the upper-right corners of each gate indicate the percentage of cells detected by the antibody. Data are representative of two independent experiments.


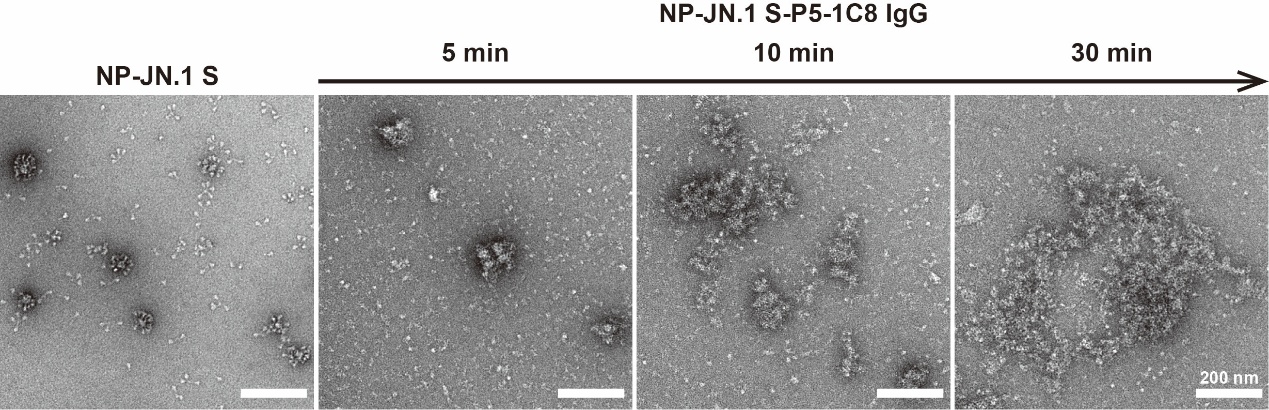
**Figure S14.** Negative-stain EM images of P5-1C8 IgG in complex with NP-JN.1 S nanoparticles after incubation for 5 to 30 min, with IgG concentration of 1.4 μM and ~0.5 μM spike trimer displayed on nanoparticles. Scale bar: 200 nm. The scale bar in the final image is representative of all images in the panel.


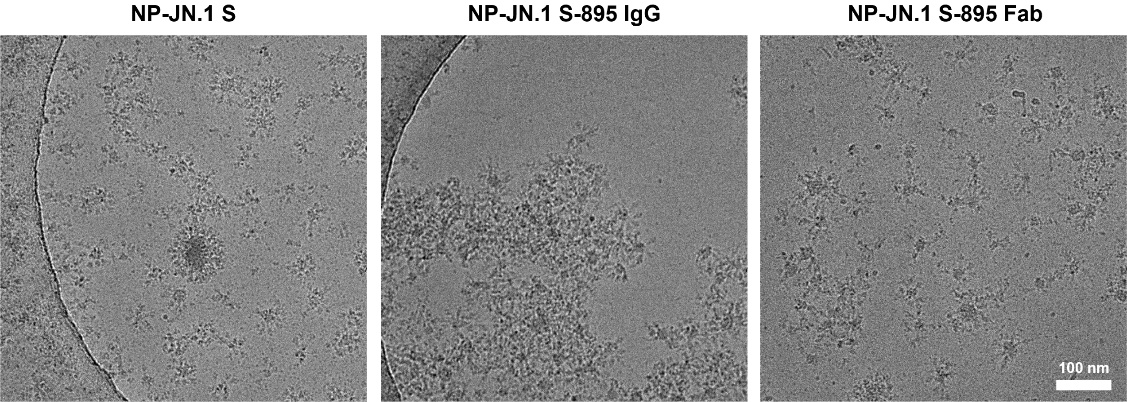
**Figure S15.** Cryo-EM images of P5-1C8 IgG or Fab in complex with NP-JN.1 S nanoparticles at an IgG concentration of 0.2 μM, with ~0.5 μM spike trimer displayed on nanoparticles. Scale bar: 100 nm. The scale bar in the final image represents all images in the panel.


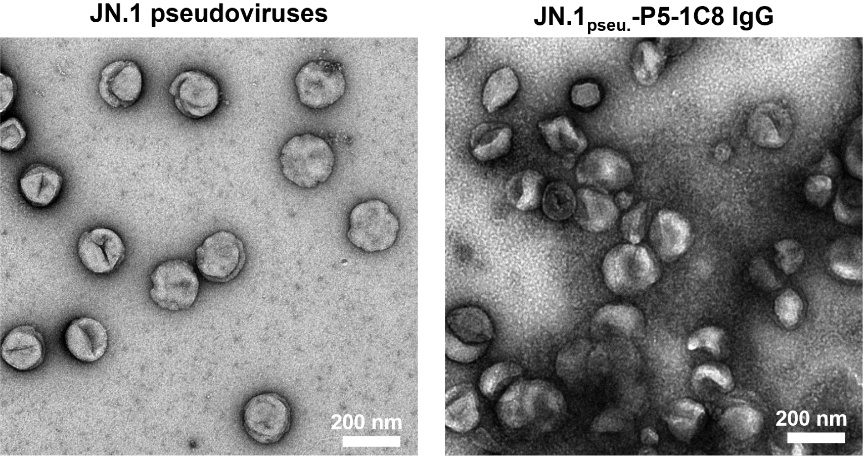
**Figure S16.** Representative negative-stain EM images of JN.1 pseudoviruses and JN.1_pseu._-P5-1C8 IgG complex. Scale bar: 200 nm.


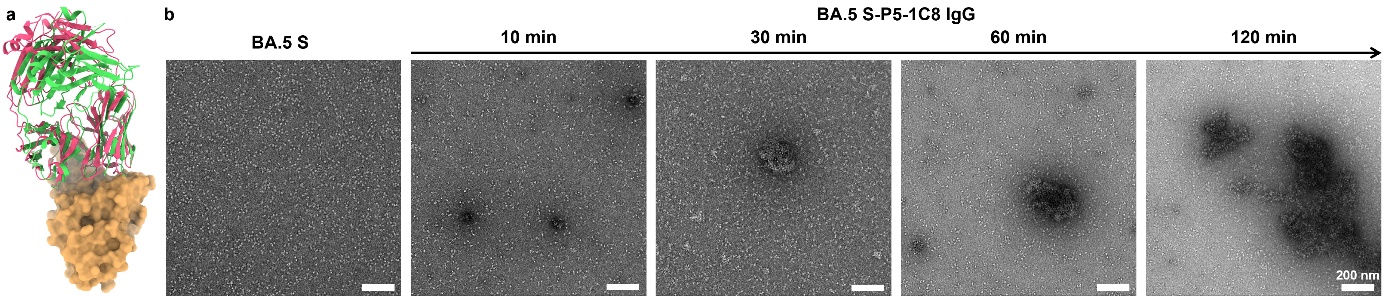
**Figure S17.** Structural comparison and negative-stain EM of P5-1H1 and BA.5 spike trimers interactions. a) Superimposed structures of P5-1H1 Fab (green) and P5-1C8 Fab (pink) bound to the SARS-CoV-2 RBD (soft orange), revealing highly overlapping epitopes and nearly identical approach angles (P5-1H1: PDB 7XS8; P5-1C8: PDB 9K6J). b) Representative negative-stain EM images of P5-1C8 IgG complexed with BA.5 spike trimers after incubation for 10 to 120 mins. Scale bar: 200 nm. The scale bar in the last image applies to all other panels.


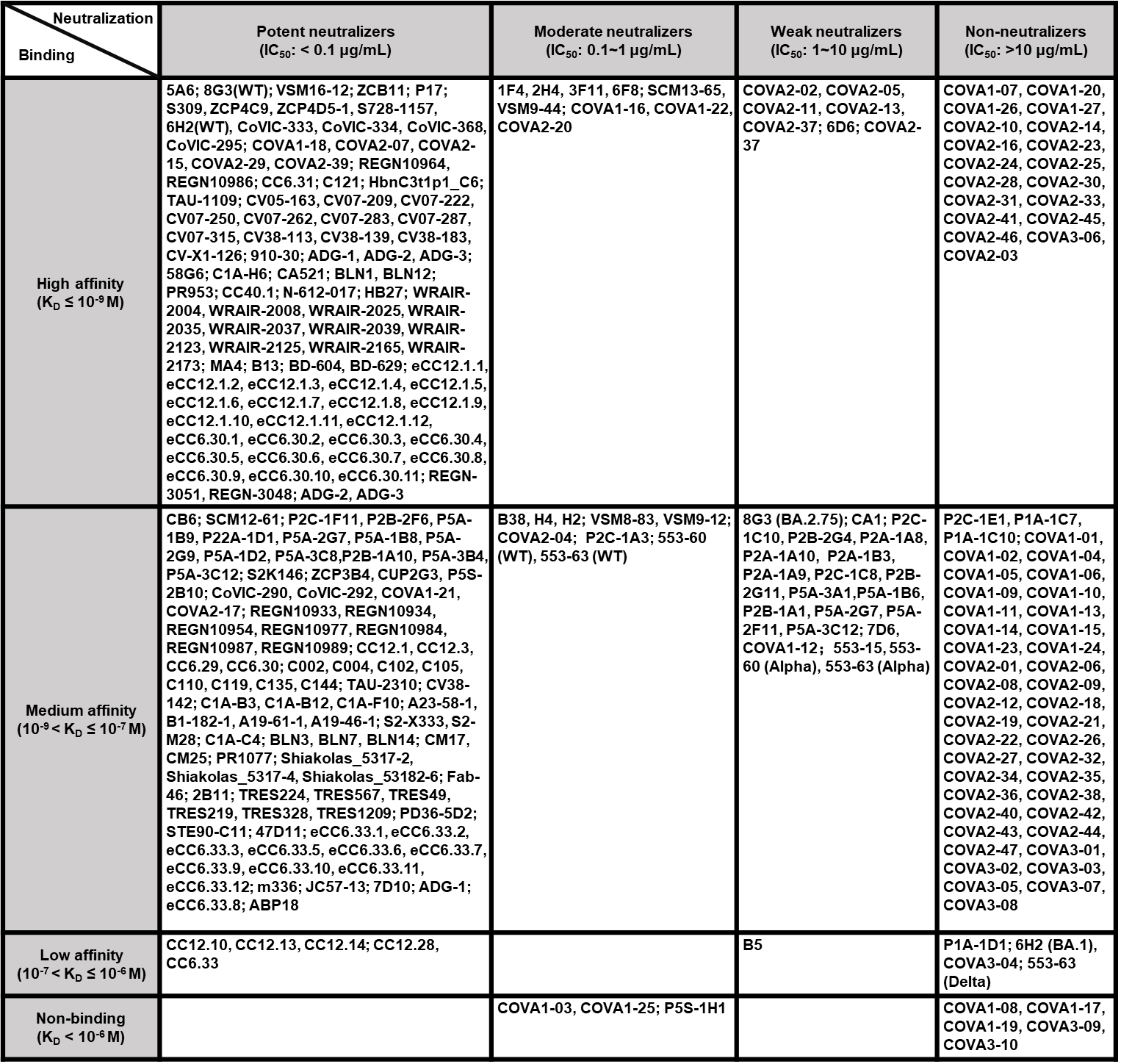
**Table S1.** Correlation of binding affinity and neutralization potency for previously reported SARS‑CoV‑2 antibodies. K_D_ values (measured by SPR or BLI) and IC_50_ values were sourced from the Ab‑CoV database (antibodies reported prior to 2022)^[1]^ and from peer‑reviewed publications in recent years. Antibodies are categorized by affinity^[2]^—high (K_D_ ≤ 10⁻⁹ M), medium (10⁻⁹ < K_D_ ≤ 10⁻⁷ M), low (10⁻⁷ < K_D_ ≤ 10⁻⁶ M), and non‑binder (K_D_ > 10⁻⁶ M or undetectable)—and by neutralization potency^[3]^—potent (IC_50_ < 0.1 μg/mL), moderate (0.1-1 μg/mL), weak (1-10 μg/mL), and non‑neutralizing (IC_50_ > 10 μg/mL).

**Table S2.** SDS-PAGE gels, size-exclusion chromatography profiles, and negative-stain EM micrographs of purified IgG, Fab, spike proteins, and RBD. N.A.: not available.


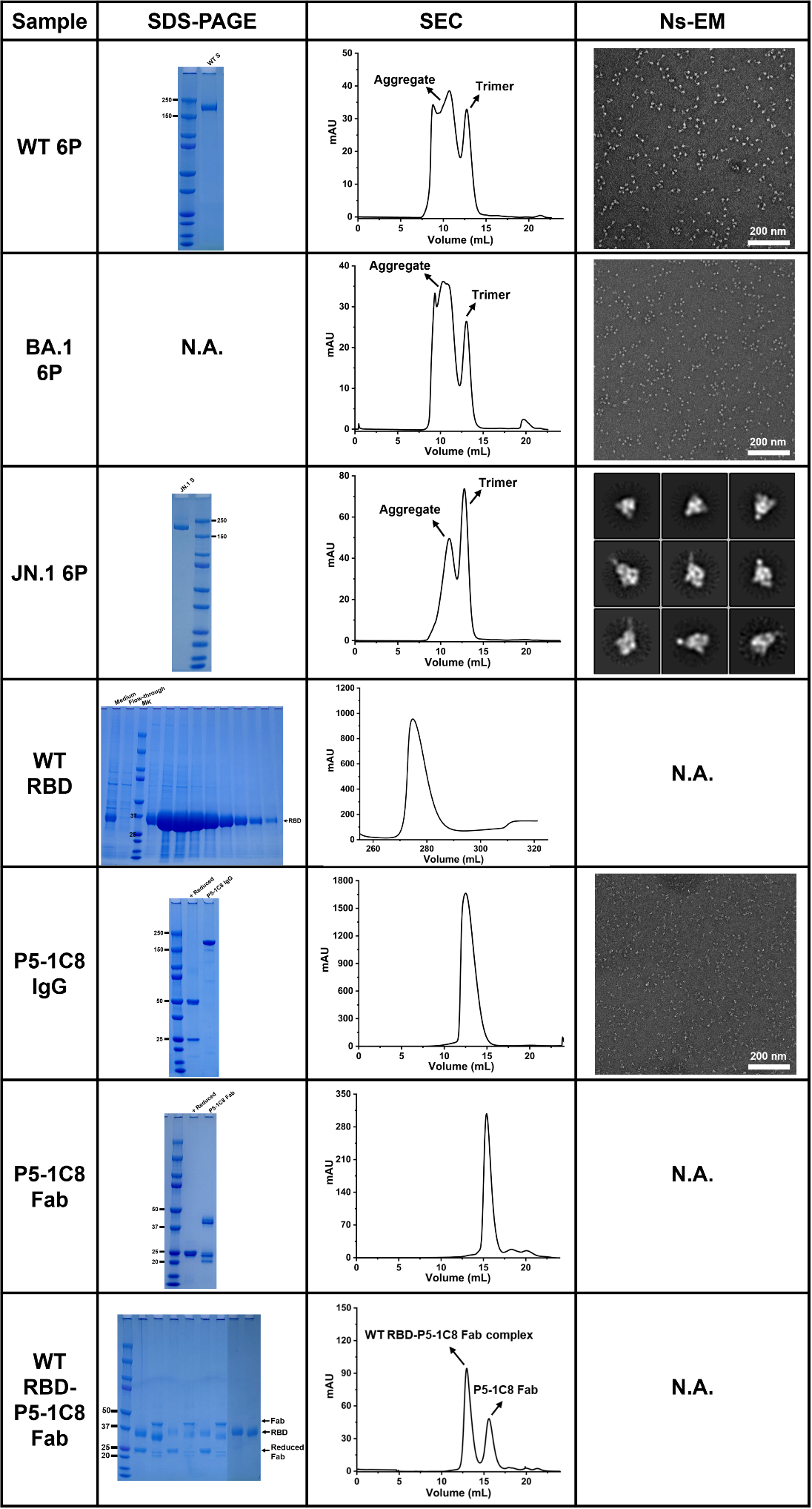


**Table S3.** Negative‑stain EM dataset summary of antibody-spike complexes and JN.1 spike-only. Entries include construct names (with EMD accession numbers), defined structural classes, particle numbers per class, raw micrographs, 2D class average, and 3D reconstruction. N.A.: not available.


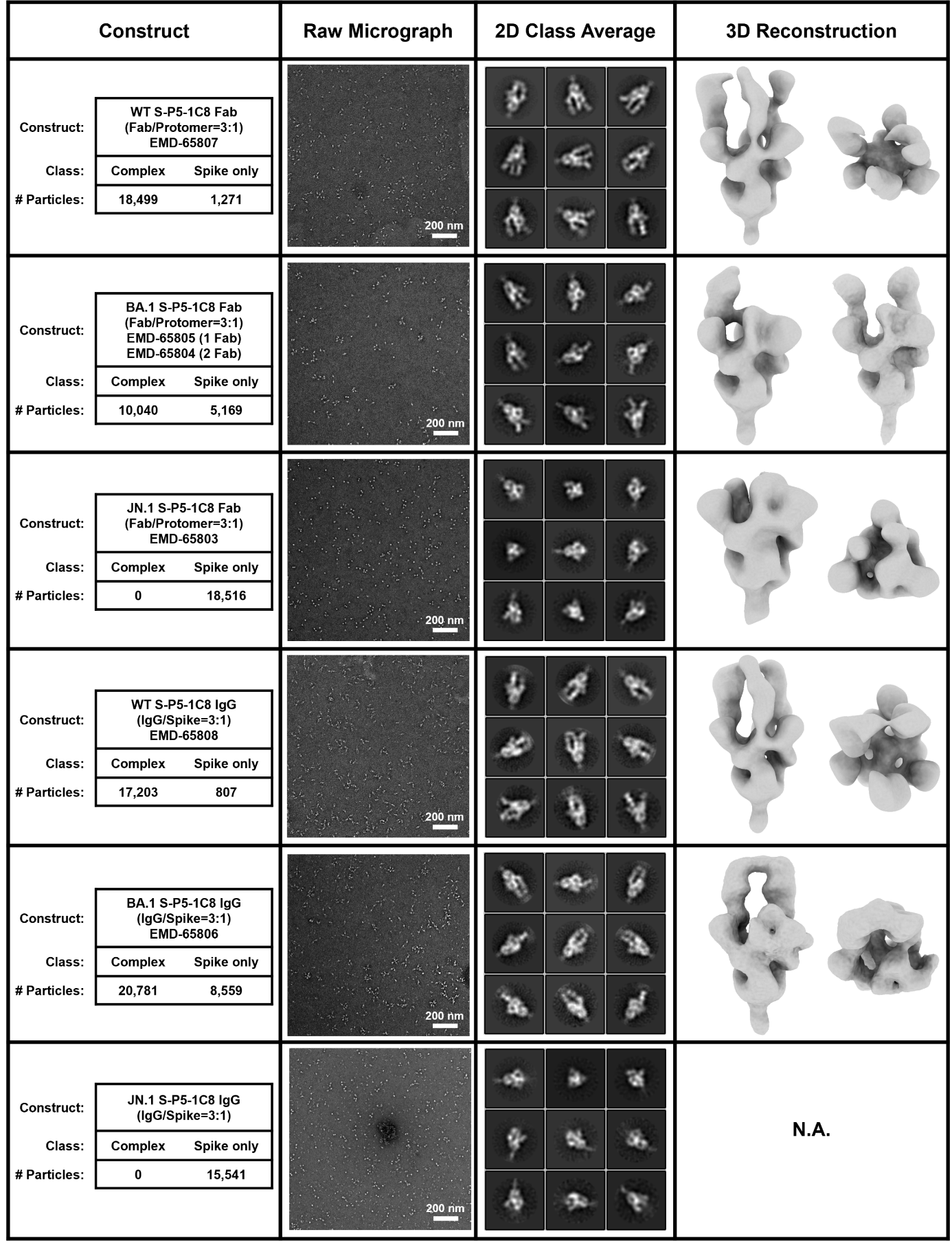


**Table S4.** The hydrogen bonds and salt bridges at the SARS-CoV-2 WT RBD and P5-1C8 Fab interfaces (distance cutoff 4 Å).

| **Hydrogen bonds** | | | |
| --- | --- | --- | --- |
| RBD | H chain | RBD | L chain |
| A475-O | I28-N | K417-NZ | Y33-OH |
| A475-O | N32-ND2 |  |  |
| L455-O | Y33-OH |  |  |
| Y421-OH | S53-N |  |  |
| R457-O | S53-OG |  |  |
| Y421-OH | G54-N |  |  |
| D420-OD2 | S56-OG |  |  |
| N487-OD1 | R97-NH1 |  |  |
| N487-OD1 | R97-NH2 |  |  |
| Y453-OH | Y102-OH |  |  |
| K417-NZ | D101-OD1 |  |  |
| K417-NZ | D101-OD2 |  |  |
| Y473-OH | R31-O |  |  |
| Q493-NE2 | Y99-OH |  |  |
| **Salt bridges** | |  |  |
| RBD | H chain |  |  |
| K417-NZ | D101-OD1 |  |  |
| K417-NZ | D101-OD2 |  |  |

Table S5. Crystallization kits are utilized during initial screening trials.

| **Swissci-Plate 96-3** | **Kit Name** | **Kit Brand** |
| --- | --- | --- |
| S-3 | Crystal Screen 1/2 | Hampton Research |
| S-4 | PEG Rx 1/2 | Hampton Research |
| S-6 | Index | Hampton Research |
| S-7 | PEG/Ion 1/2 | Hampton Research |

**Table S6.** Cryo-EM Data collection parameters of SARS-CoV-2 WT S-P5-1C8 IgG complexes.

| Sample | SARS-CoV-2 WT S-P5-1C8 IgG  EMD-65801 (1.5 IgG)  EMD-65802 (1 IgG) |
| --- | --- |
| **Data collection and processing** |  |
| Magnification | 29000x |
| Voltage (kV) | 300 |
| Electron exposure (e^-^/Å^2^) | 50 |
| Defocus range (μm) | -1 to -1.6 |
| Pixel size (Å) | 0.97 |
| Micrographs (No.) | 940 |
| Initial particles (No.) | 328,106 |
| Final particles (No.) | 152,697 |
| Map resolution (Å) | 5.48 |
| Local resolution range (Å) | 7.75 |
| Map sharpening B-factor (Å^2^) | 302.9 |

**Table S7.** Data collection and refinement statistics (molecular replacement).

|  | SARS-CoV-2 WT RBD & P5-1C8 Fab  (PDB-9K6J) |
| --- | --- |
| **Data collection** |  |
| Space group | P 21 21 21 |
| Unit cell dimensions |  |
| *a, b, c* (Å) | 55.306 115.092 144.135 |
| *α, β, γ* (°) | 90.0 90.0 90.0 |
| Resolution range (Å) | 89.94 - 2.39 (2.52 - 2.39) |
| Rmerge (%) | 0.459 (1.537) |
| *I/σ I* | 4.400 (1.500) |
| Completeness (%) | 100.0 (100.0) |
| Redundancy | 9.7 (9.0) |
| CC1/2 | 0.951 (0.413) |
|  |  |
| **Refinement** |  |
| Resolution (Å) | 53.44-2.39 |
| No. reflections | 34149 (3575) |
| *Rwork/Rfree* (%) | 23.58/28.29 |
| No. atoms |  |
| Protein | 4611 |
| Ligands | 14 |
| Water | 157 |
| *B-factor* (Å^2^) |  |
| Protein | 25.78 |
| Ligands | 61.87 |
| Water | 21.76 |
| R.m.s deviations |  |
| Bond length (Å) | 0.292 |
| Bond angles (°) | 0.465 |
| Ramachandran plot |  |
| Favored (%) | 96.98 |
| Allowed (%) | 3.02 |
| Outliers (%) | 0.00 |

**Table S8.** Summary of coarse-grained MD simulation parameters as deﬁned in the Supporting Information. All simulations were conducted with spike-decorated nanoparticles (NPs) bearing 8 spike trimers per NP to align with experimental conditions.

| **Category** | **Parameter** | **Value / Setting** | **Source (SI)** |
| --- | --- | --- | --- |
| Unit  System | Length unit (σ) | 7*.*5 nm | Eq. (1) |
|  | Time unit (t_0_) | 0*.*1 µs | Eq. (1) |
|  | Energy unit (ε) | *k_B_T* at 300 K = 4*.*14 × 10*^−^*^21^ J | Eq. (1) |
| Molecular  Dimensions | Nanoparticle radius (R_np_) | 7*.*5 nm | Text |
|  | Spike length (L_spike_) | 25 nm | Text |
|  | IgG length (L_IgG_) | 15 nm | Text |
| Molecular  Masses | Spike mass (M_Spike_) | 540 kg*/*mol | Text |
|  | IgG mass (M_IgG_) | 150 kg*/*mol | Text |
| Concentrations | Spike on NP | 0*.*5 µM (8 spike trimers per NP) | Text |
|  | IgG | 1*.*4 µM | Text |
| Simulation  Setup | Software | GALAMOST | Ref. [43] |
|  | Ensemble | NVT | Text |
|  | Thermostat | Velocity rescaling  (τ = 0*.*2 ps) | Text |
|  | Barostat | Nose-Hoover  (τ = 1*.*0 ps, parameter = 0.5) | Text |
|  | Time step | 4 ns | Text |
|  | Total duration | 15 µs | Text |
|  | Box size | 1500×1500×1500 nm^3^ | Text |
|  | Trajectory save interval | Every 1 ns | Text |
| Interaction  Potentials | Bonded | Harmonic bond/angle + dihedrals | Eqs.(2), (3) |
|  | Non-bonded | LJ + Coulomb (reaction ﬁeld) | Eqs.(4), (5) |
|  | Force Field | MARTINI parameterization | Text |

**Legends for Movies S1 to S2:**

**Movie S1.** Coarse-grained molecular dynamics simulation of NP-WT S nanoparticles interacting with full-length P5-1C8 IgG. The system contains 1.4 μM IgG and ~0.5 μM WT spike trimer displayed on nanoparticles. Nanoparticles are shown in grey. WT spikes are segmented by amino-acid numbering into four domains—residues 1-527 (NTD and RBD), 528-833 (CTD1, CTD2, and FP), 834-1162 (HR1, CH, and CD), and 1163-1240 (HR2 and foldon)—and rendered as a continues blue gradient from top (intense blue) to bottom (faded blue). P5-1C8 IgG is shown in pink, with both Fab domains in pink and the hinge region and Fc domain in pale pink.

**Movie S2.** Coarse-grained molecular dynamics simulation of NP-JN.1 S nanoparticles interacting with full-length P5-1C8 IgG. The system contains 1.4 μM IgG and ~0.5 μM JN.1 spike trimer displayed on nanoparticles. Nanoparticles are shown in grey. JN.1 spike are segmented by amino-acid numbering into four domains—residues 1-527 (NTD and RBD), 528-833 (CTD1, CTD2, and FP), 834-1162 (HR1, CH, and CD), and 1163-1240 (HR2 and foldon)—and rendered as a continues green gradient from top (intense green) to bottom (faded green). P5-1C8 IgG is shown in pink: both Fab domains are pink while the hinge region and Fc domain are rendered in pale pink.

**Reference**

[1] P. Rawat, D. Sharma, R. Prabakaran, F. Ridha, M. Mohkhedkar, V. Janakiraman, M. M. Gromiha, Ab-CoV: a curated database for binding affinity and neutralization profiles of coronavirus-related antibodies. *Bioinformatics* **2022**, *38* (16), 4051.

[2] T. R. Poulsen, P.-J. Meijer, A. Jensen, L. S. Nielsen, P. S. Andersen, Kinetic, affinity, and diversity limits of human polyclonal antibody responses against tetanus toxoid. *J. Immunol.* **2007**, *179* (6), 3841.

[3] P. J. M. Brouwer, T. G. Caniels, K. van der Straten, J. L. Snitselaar, Y. Aldon, S. Bangaru, J. L. Torres, N. M. A. Okba, M. Claireaux, G. Kerster, A. E. H. Bentlage, M. M. van Haaren, D. Guerra, J. A. Burger, E. E. Schermer, K. D. Verheul, N. van der Velde, A. van der Kooi, J. van Schooten, M. J. van Breemen, T. P. L. Bijl, K. Sliepen, A. Aartse, R. Derking, I. Bontjer, N. A. Kootstra, W. J. Wiersinga, G. Vidarsson, B. L. Haagmans, A. B. Ward, G. J. de Bree, R. W. Sanders, M. J. van Gils, Potent neutralizing antibodies from COVID-19 patients define multiple targets of vulnerability. *Science* **2020**, *369* (6504), 643.
